# Cell-Membrane-Anchored Synthetic Dynamic DNA Circuits for Signaling Transient Cell Migration

**DOI:** 10.1021/jacs.5c03070

**Published:** 2025-09-12

**Authors:** Nina Lin, Yu Ouyang, Yunlong Qin, Songqin Liu, Itamar Willner, Yuanjian Zhang, Zhixin Zhou

**Affiliations:** † School of Chemistry and Chemical Engineering, 12579 Southeast University, Nanjing 211189, China; ‡ Institute of Chemistry, 26742 The Hebrew University of Jerusalem, Jerusalem 91904, Israel

## Abstract

A DNA reaction circuit consisting of components E/Q_1_, E_1_/T_1_, F/Q_1_, and F_1_/T_1_ in which each includes the antimesenchymal epithelial transition (Met) receptor aptamer sequence is anchored within MCF-7 cells to emulate the natural signaling network on the live cell membrane. Subjecting the membrane-integrated circuit to an auxiliary fuel strand, in the presence of a nicking enzyme, results in the dynamic reconfiguration of the circuit into a constitutional dynamic network, CDN, in which the pre-engineered duplex interactions between the constituents lead to allosterically stabilized Met-dimer complexes. The concomitant nickase-induced separation of the CDN leads to the parent reaction circuit, and to the transient formation and depletion of the Met-dimer complex. By labeling the components comprising the reaction circuits with fluorophores, the dynamic transient reconfiguration of the CDN and the accompanying Met-dimer formation and separation within the cell membranes are characterized by temporal confocal fluorescence microscopy imaging. Moreover, the transient formation of the Met-dimer in the MCF-7 cell membrane induces intracellular signaling and activation of the Akt/FAK phosphorylation pathway. This is reflected by the network-guided control over the transient migration/motility functions of the MCF-7 cells.

## Graphical abstract

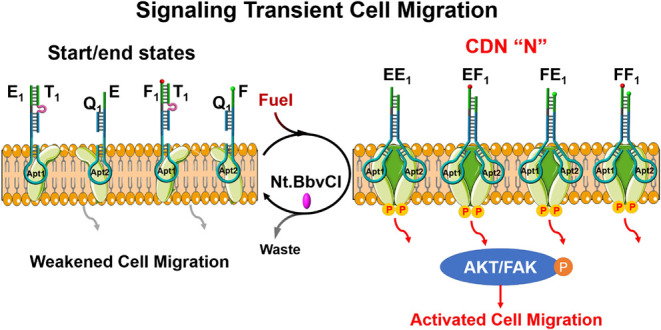

## Introduction

Communication between cells and their exterior environment is a key feature regulating physiological processes, such as differentiation,^1^ migration,^2^ apoptosis,^3^ cell intercommunication,^4–6^ and cellular metabolism.^7^ These processes are driven by spatial and temporal dynamic networks featuring sensing,^8^ triggering by external stimuli,^9^ signaling,^10^ feedback amplification,^11^ cascaded,^12^ and temporal^13^ or oscillatory pathways.^14^ Emulating biological networks by synthetic DNA networks attracts substantial recent research efforts.^15^ The programmability of base sequences encoded in DNA,^16^ and the emergent recognition features (aptamers) or catalytic properties of DNA (DNAzymes)^17,18^ provide means to synthetically engineer complex DNA-based networks. Moreover, the reversible and switchable reconfiguration of oligonucleotides by external stimuli, such as strand displacement,^19,20^ pH-responsive i-motif structures,^21^ DNA triplexes,^22^ G-quadruplexes,^23^ metal-ion-bridged mismatched duplexes,^24,25^ and light-stimulated stabilization/destabilization of duplex nucleic acids by photoisomerizable intercalators,^26^ provides a rich arsenal of structural motifs for the construction of adaptive, dynamically reconfigurable, DNA-based networks. Indeed, these features of DNA were implemented to develop synthetic chemical reaction networks,^27^ neural networks,^28^ and constitutional dynamic networks (CDNs)^29^ modeling features of biological circuits. In particular, DNA-based CDNs attracted substantial recent research efforts, and CDNs triggered by triplexes^30^ or light,^31^ revealing hierarchically adaptative^32^ and feedback-driven pathways^33^ were demonstrated. Different applications of CDNs were reported, including their use as functional dynamic frameworks guiding biocatalytic cascades,^34^ dynamic networks for gene therapy,^35^ and the fabrication of dynamic soft hydrogel materials.^36^

In addition, the development of out-of-equilibrium dynamic dissipative, transient, DNA circuits attracts growing interest.^37,38^ In these systems, a mute reaction module is activated by a fuel agent that yields a metastable intermediate product. The reaction module includes, however, a built-in chemical or physical agent that degrades the intermediate, thereby recovering the parent reaction module, while generating “waste” products. Different triggers, including DNA strands,^39^ chemical fuels,^40^ and light,^41^ were applied to activate the transient circuits, and enzymes,^42^ DNAzymes,^43^ or nonenzymatic chemical reaction^44^ were used to deplete the intermediate products and transiently recover the inactive parent reaction modules. Different applications of transient DNA reaction circuits were reported, including the transient operation of aggregation/deaggregation of particles, catalytic functions,^45–47^ or the formation/dissolution of DNA-based microdroplets/fibers.^48,49^

Nevertheless, the different transient dynamic networks and circuits were operated in homogeneous aqueous phases. The challenges of Systems Chemistry^50,51^ and Systems Biology^52,53^ include, however, the integration of the dynamic regulatory networks within living cells mimicking the signaling network on the cell membrane and the temporal, transient activation of cell functions within the hybrid composites. Reaching these goals would require the engineering of dynamic network within the cell environments, the design of stimuli-driven, transient pathways signaling the cell toward target functions, and the development of analytical tools to characterize the systems and monitor the network-dictated cell functions. Indeed, substantial recent research efforts were directed toward the engineering of synthetic phosphorylation signaling networks in cells,^10^ and the significance of phosphor-signaling circuits for autonomous sense-and-response therapies has been discussed.^54^ In addition, recent studies reported on the integration of DNA circuits within cell membrane boundaries, with the vision that such circuits could signal intracellular functions. For example, a DNA walker circuit was incorporated into cell membranes as a molecular robot that walked on the cell membrane to signal cell motility.^2^ Nevertheless, this walker system lacked switchable or reversible functionalities. Also, photoswitchable dimerization and separation of the receptor unit signaling intracellular phosphorylation pathways using azobenzene-modified aptamers were reported.^55^ However, the subsequent cell motility function and the transient signaling operation by the photoactive, membrane-bound circuit were not demonstrated. In fact, the integration of synthetic dissipative dynamic circuits within cell membranes signaling switchable, transient cell functions such as cell migration and motility is unprecedented. The development of such systems is anticipated to introduce revolutionizing concepts to the fields of Systems Chemistry and Systems Biology and have an enormous impact on developing new therapeutic pathways.

Here we wish to report on the integration of synthetic nucleic acid circuits within the cell membrane. The signal-triggered reconfiguration of the circuits into transiently operating CDNs, within the cell membranes, is demonstrated. By one approach, a reaction module consisting of caged cholesterol-modified nucleic acid strands is embedded in Human Embryonic Kidney 293T (HEK-293T) cell membranes to emulate the natural signaling network. The unlocking of the reaction circuit by a fuel strand, in the presence of nickase as an auxiliary agent, reconfigures the reaction circuit into a transiently operating CDN. The dynamic process is followed by temporal confocal fluorescence microscopy imaging and flow cytometry experiments. A second approach involves the application of a reaction circuit composed of caged nucleic acid strands that include the Met-aptamer. The caged reaction circuit is integrated within the MCF-7 cell membrane by the Met receptor in the cell membrane. In the presence of an auxiliary fuel strand and nickase as a trigger, the reconfiguration of the circuit into a temporally operating CDN is demonstrated. The intramembrane evolution of the CDN yields spatially proximate Met-receptor/Met-aptamer complexes costabilized by cooperative allosteric base-pairing interactions within the nucleic acid constituents. The formation of the Met dimer provides intracellular phosphorylation signaling activating the Akt/FAK pathway toward cell migration/motility.^56,57^ The temporal formation and transient depletion of the CDN framework within the cell membrane is then translated by the triggered transient migration functions of the cells. While transient, dissipative circuits were extensively studied in homogeneous aqueous phases, their integration into native cell membranes and the sequestered temporal signaling of cell functions are unprecedented. The main accomplishment of the present study is reflected by the integration of a synthetic dissipative reaction circuit into native membranes signaling intracellular cell functionalities. The concept of intercommunication between the synthetic circuit and the cell signaling pathway, using a programmable aptamer/receptor complex, and the application of a set of transiently equilibrated four constituents, could allow future operation of gated signaling intracellular pathways. We anticipate that the key discovery of controlling cell functions by synthetic DNA circuits embedded in the cell membrane could be extended to other functional synthetic systems/cell hybrid configurations that could be adapted for different therapeutic applications.

## Resulsts and Discussion

The schematic composition and mode of operation of a synthetic transient CDN planned to be integrated in the cell membrane are displayed in Figure 1A. The “rest” reaction module is composed of A_1_/T_1_, B_1_/T_1_, A/Q, and B/Q duplexes (quencher-functionalized Q-strand) and the nicking enzyme (Nt.BbvCI). Each of the components A_1_, B_1_, A, and B includes a Mg^2+^-dependent DNAzyme subunit, and components A and B are modified with fluorophores Cy3 in a quenched configuration by a Q-strand. Subjecting the “rest” module to the fuel strand T_1_′ results in the displacement of duplexes A_1_/T_1_ and B_1_/T_1_, yielding free A_1_, B_1_, and concomitant duplex T_1_/T_1_′. The released strands A_1_ and B_1_ displace the A/Q and B/Q duplexes, resulting in the emergence of four duplex constituents, AA_1_, AB_1_, BA_1_, and BB_1_ that comprise CDN “K”. The evolved CDN “K” includes in each of the constituents an emergent, yet different, built-in, Mg^2+^-dependent DNAzyme unit (different functional “arms”) that acts as reporter units probing the concentrations of the respective constituents through the DNAzyme-catalyzed cleavage of the respective fluorophore/quencher (F_i_/Q_i_)-modified substrates. Concomitant to the emergence of CDN “K”, the displaced T_1_/T_1_′ duplex is pre-engineered to include in T_1_′ the sequence-specific nicking site to be cleaved by the nickase. Cleavage of T_1_′ yields fragmented, separated, “waste” products T_1–1_′ and T_1–2_′, and free T_1_ that displaces, together with the free Q-strand, the constituents of CDN “K”, thus leading to the transient recovery of the “rest”, parent, reaction module. The transient formation/depletion of CDN “K” is then probed by the temporal fluorescence changes associated with constituents (AB_1_ + AA_1_) and (BA_1_ + BB_1_), and the kinetic features of the DNAzyme reporter units linked to constituents AA_1_, AB_1_, BA_1_, and BB_1_. For the Gibbs free energy balance accompanying the transient formation of CDN “K” and the favored energy-driven recovery of the parent reaction module, upon the nickase cleavage of T_1_′/T_1_ and generation of the waste products, see Figure S1 and accompanying discussion.

**Figure 1:**
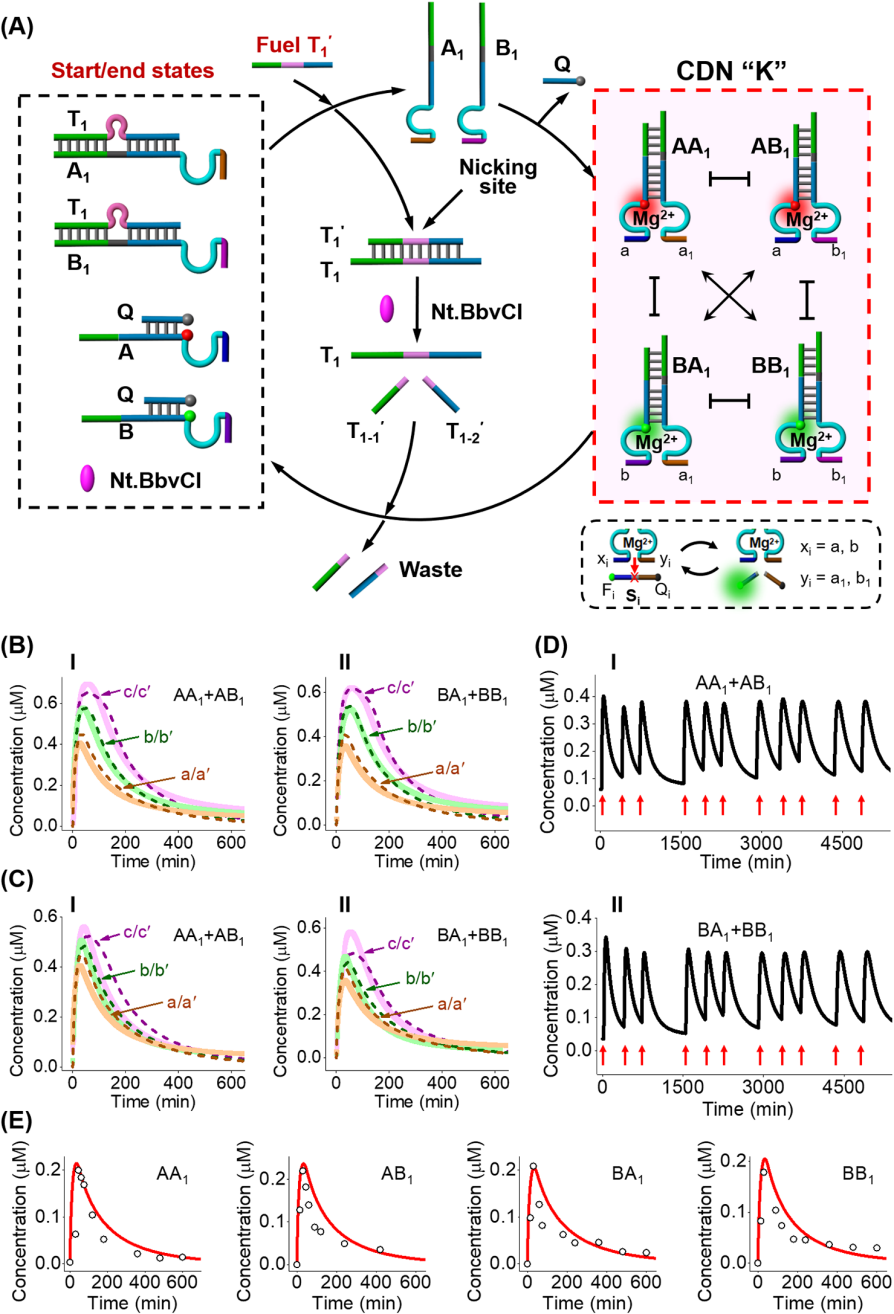
Fuel-driven reconfiguration of a reaction module into a transient operating constitutional dynamic network. (A) A reaction module consisting of four components and a nicking enzyme, Nt.BbvCI, leading in the presence of a fuel strand, T_1_′, to the transient evolution of a CDN “K”. (B) Temporal concentrations of the constituents (AA_1_ + AB_1_), Panel I, and (BA_1_ + BB_1_), Panel II, upon the T_1_′-triggered, transient, formation and depletion of CDN “K”, in the presence of different concentrations of the fuel strand T_1_′: (a/a′) 3 μM, (b/b′) 5 μM, and (c/c′) 8 μM. (C) Temporal concentrations of the constituents (AA_1_ + AB_1_), Panel I, and (BA_1_ + BB_1_), Panel II, upon the T_1_′-triggered, transient formation and depletion of CDN “K”, in the presence of different concentrations of the nicking enzyme: (a/a′) 0.069 μM, (b/b′) 0.046 μM, and (c/c′) 0.023 μM. (a/b/c, solid curves, experimental results; a′/b′/c′, dashed curves, computational results are detailed in Figures S4 and S5). (D) Cyclic T_1_′-triggered, transient, temporal concentrations of constituents (AA_1_ + AB_1_), Panel I, and constituents (BA_1_ + BB_1_), Panel II, upon repeated T_1_′-triggered activation of evolution/depletion cycles of CDN “K”, in the presence of Nt.BbvCI, 0.069 μM. (E) Transient concentration changes of the constituents in CDN “K” evaluated by DNAzyme reporter units upon the T_1_′-triggered transient evolution of the CDN “K” in the presence of T_1_′, 3 μM, and nickase, 0.069 μM. (For the computational simulations of the transient behaviors of the system, see Figures S4 and S5.).

Figure 1B depicts the transient concentrations of (AA_1_ + AB_1_), Panel I, and of (BA_1_ + BB_1_), Panel II, in the presence of variable concentrations of T_1_′, evaluated by following the fluorescence changes of Cy3 and using appropriate calibration curves (Figure S2). Figure 1C shows the transient concentrations of (AA_1_ + AB_1_), Panel I, and of (BA_1_ + BB_1_), Panel II, in the presence of variable concentrations of nickase, evaluated by following the fluorescence changes of Cy3 and using appropriate calibration curves. For additional experiments probing the effects of variable concentrations of the fuel strand T_1_′ and the concentrations of nickase on the transient evolution of CDN “K” and nickase-driven depletion of CDN “K” to the parent constituents, see Figure S3. Figure 1D demonstrates the transient, fueled emergence of CDN “K” and the nickase-stimulated depletion of the network and the capacity to recycle the dissipative process by readdition of the fuel T_1_′. Figure 1E depicts the transient concentration changes of the constituents transduced by the DNAzyme reporter units (for details, see Figures S6–S9 and Table S1). In addition, the T_1_′-induced dissipative evolution of CDN “K” was supported by quantitative gel electrophoresis, Figure S10, and accompanying discussion.

The concept of dynamically fueled, triggered operation of a transient formation/depletion of a CDN was then adapted to assemble a cell-membrane-integrated reaction module operating as a CDN within the membrane boundary mimicking natural signaling networks, Figure 2. A set of cholesterol-modified components (chol) consisting of C_1_-chol/T_1_, D_1_-chol/T_1_, C-chol/Q-strand, and D-chol/Q-strand was integrated in HEK-293T cell membranes, where strands C and D were labeled with internal fluorophores Cy3 and Cy5 in a fluorescence-quenched configuration. In addition, each of the components, C, C_1_, D, and D_1_, is functionalized with a sequence composed of the DNAzyme subunit. Subjecting the reaction module integrated in the cell membrane to fuel strand T_1_′ and auxiliary nickase (Nt.BbvCI) results in the displacement of components C_1_/T_1_ and D_1_/T_1_, yielding the duplex T_1_/T_1_′, and the released components C_1_ and D_1_. The free C_1_ and D_1_ displace components C/Q-strand and D/Q-strand to generate the membrane-integrated CDN “M”. The concomitant nickase-stimulated cleavage of the duplex T_1_/T_1_′ releases T_1_, leading to the temporal displacement of the constituents in CDN “M” and the recovery of the parent, membrane-distributed reaction module. The transient and cyclic dynamic emergence and depletion of the membrane-linked CDN “M” constituents were, then, probed by the temporal fluorescence intensity changes of fluorophores Cy3 and Cy5 associated with constituents (CC_1_ + CD_1_) and (DD_1_ + DC_1_), respectively, and the temporal catalytic responses of the DNAzyme reporters associated with the constituents CC_1_, CD_1_, DC_1_, and DD_1_. (Note that for the assembly of the DNA circuit on the cell membrane, high concentrations of C/Q, C_1_/T_1_, D/Q, and D_1_/T_1_ were used due to the low-affinity binding of the cholesterol-modified constituents to the membrane. For a detailed procedure to assemble the cholesterol-modified nucleic acid circuit on the cell membrane and optimization of binding of the constituents to the membrane, see p.S4 to p.S5, Figures S11 and S12, and the accompanying discussion.)

**Figure 2:**
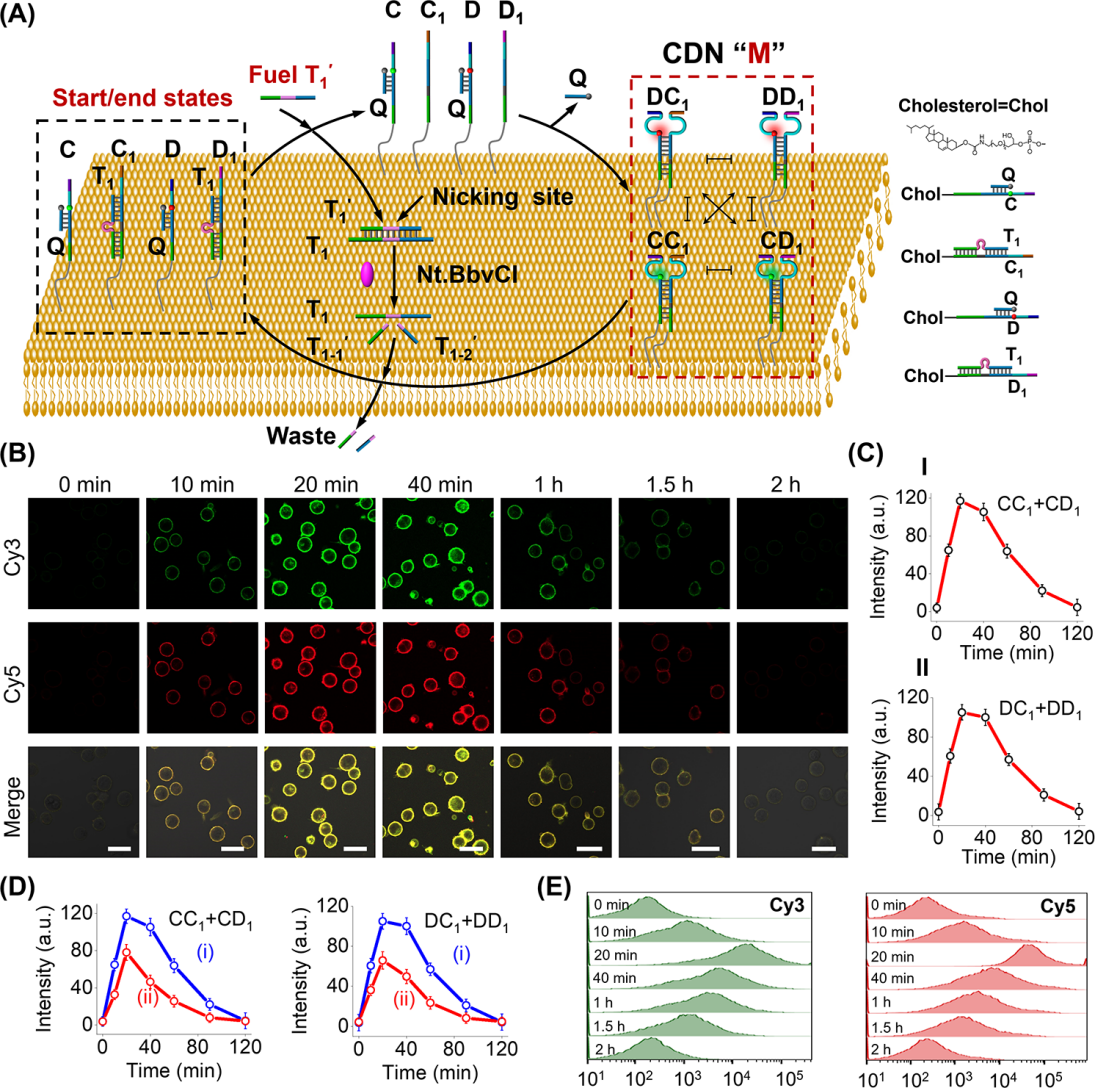
Fuel-driven reconfiguration of a cell membrane-anchored oligonucleotide reaction module into a transient operation of constitutional dynamic network. (A) Schematic T_1_′-triggered dynamic transient reconfiguration of a reaction module consisting of cholesterol-modified components C/Q, C_1_/T_1_, D/Q, and D_1_/T_1_, associated within a HEK-293T cell membrane into CDN “M”, in the presence of Nt.BbvCI as an auxiliary agent. (B) Temporal confocal fluorescence microscopy images corresponding to fluorophore Cy3 (green) associated with the constituents (CC_1_ + CD_1_), fluorophore Cy5 (red) associated with the constituents (DC_1_ + DD_1_), and bright-field overlay (yellow), upon the T_1_′-triggered evolution/depletion of CDN “M”, in the presence of T_1_′, 100 nM. Scale bar: 20 μm. (For high-resolution images of cell morphologies, see Figure S12.) (C) Transient integrated confocal fluorescence intensities, upon T_1_′ (100 nM)-triggered evolution/depletion of CDN “M” probed at: Panel I-constituents (CC_1_ + CD_1_), and Panel II-constituents (DC_1_ + DD_1_). (D) Transient integrated fluorescence intensities of constituents (CC_1_ + CD_1_) and constituents (DC_1_ + DD_1_) upon evolution/depletion of CDN “M” in the presence of: (i) T_1_′ = 100 nM, (ii) T_1_′ = 30 nM, and nickase. (E) Temporal integrated fluorescence intensities of cell samples probed by flow cytometry using the Cy3 channel and Cy5 channel in the presence of the trigger T_1_′ and nickase.

Figure 2B depicts the temporal confocal fluorescence images of the cells using the green Cy3 fluorophore associated with (CC_1_ + CD_1_) and the red Cy5 fluorophore associated with (DC_1_ + DD_1_). Figure 2B presents the temporal fluorescence images of the cells, upon fueling the dynamic reconfiguration of CDN “M” in the presence of the fuel strand, T_1_′, 100 nM, whereas Figure S13 shows the temporal two-channel fluorescence images of the cells, in the presence of T_1_′, 30 nM. The merged yellow images are, also, provided. The temporal fluorescence changes upon the T_1_′-triggered formation of the CDN associated with the cells reveal an initial increase in the green/red fluorescence intensities, followed by a transient depletion of the fluorescence. For the control system without fuel T_1_′, only a negligible increase in green and red fluorescence intensities on the membrane surface was observed over a period of hours (Figure S14). These results are consistent with the T_1_′-triggered reconfiguration of the reaction module into the CDN “M” that undergoes subsequent depletion and recovery into the parent reaction module, due to the nickase-stimulated cleavage of T_1_′ in the T_1_′/T_1_ duplex and the reverse separation of the constituents by the released T_1_. The apparent transient assembly of CDN “M” on the cell membrane was further evaluated by monitoring the temporal integrated fluorescence intensities obtained in the respective frames. The results are displayed in Figure 2C, demonstrating the temporal transient features of constituents (CC_1_ + CD_1_) and (DC_1_ + DD_1_) associated with CDN “M” linked to the cell membrane. Moreover, the results presented in Figure S15 indicate that by cyclic addition of the fuel strand T_1_′, the cyclic transient operation of CDN “M” within the cell membrane proceeds. Moreover, the transient nickase-induced recovery of CDN “M” to the parent separated reaction module is specific for nickase, Nt.BbvCI, and other nickases or endonucleases have no effect on the depletion of CDN “M” (see Figure S16 and accompanying discussion). (Experiments evaluating the stability and reusability of the constituents and dynamic reusability of the network are presented in Figures S17 and S18 and accompanying discussion.) We find that the CDN “M” network can be recycled in the presence of the cell culturing media for at least four repeated cycles with no observable degradation of the network composition or its dynamic operation.

Moreover, the dynamic transient signaling network associated with the cell membrane can be controlled by the concentration of fuel strand T_1_′ and the concentration of nicking enzyme. Figures 2D and S19 indicate that increasing the concentration of T_1_′ prolongs the transient lifetime of the CDN constituents. This is consistent with the higher T_1_′-stimulated content of the duplex T_1_/T_1_′ that, in the presence of a fixed concentration of the nickase, requires longer time intervals to be depleted. Furthermore, Figure S19 presents the effect of nickase concentrations on the dynamics of the CDN associated with the cell membrane. In addition, the dynamic T_1_′-triggered transformation of components C_1_/T_1_, D_1_/T_1_, C/Q, and D/Q into CDN “M” and the nickase-induced transient reconfiguration of CDN “M” to the “Rest” state were further probed by flow cytometry. Figure 2E presents the temporal integrated fluorescence intensities of Cy3 and Cy5 in the presence of fuel strand T_1_′. Evidently, the reconfigured constituents of CDN “M” reveal dissipative transient behaviors that correlate well with the temporal fluorescence confocal microscopy results. The DNAzyme reporter units conjugated to the constituents allow us to follow the changes of the constituents, along the dynamic T_1_′-triggered reconfiguration of the reaction module into CDN “M” and the nickase-induced separation of CDN “M” into the parent components (see Figure S20 and accompanying discussion). Furthermore, the potential cytotoxicity of CDN “M” on the HEK-293T cell was examined, Figure S21A. No effect on the cell viability was observed.

The results demonstrate the integration of a synthetic DNA circuit into the cell membrane, the successful triggered dynamic reconfiguration of the circuit into a CDN, and the transient biocatalysis-induced recovery of the CDN into the parent DNA reaction circuit. Moreover, the study provided experimental tools to follow the dynamic processes of the synthetic circuits within the membrane. These accomplishments encouraged us to try and implement a membrane-embedded dynamic network to signal controlled cell functions, and these efforts will be addressed in the subsequent section. Cellular metabolic and homeostatic pathways are modulated by spatial and temporal dynamics of diverse auxiliary signaling networks.^56^ Particularly, cell membrane-associated receptors responsive to extracellular chemical or physical stimuli, such as receptor tyrosine kinases (RTK),^58^ T-cell receptors (TCR),^59^ or G-protein couple receptors (GPCR),^60^ stimulate intracellular physiological and pathological processes in time and space, including metabolism, proliferation, differentiation, migration, and apoptosis. Moreover, often, dynamic oligomerization or deaggregation of membrane protein receptors plays key roles in activating downstream signaling cascades for operating intracellular pathways. Inspired by nature, we attempted to integrate a synthetic dissipative transient CDN within MCF-7 cells for the controlled dynamic reconfiguration of the Met receptor, resulting in CDN-guided downstream signaling and triggered transient motility functions. The Met receptor binds the hepatocyte growth factor ligand that, upon triggered dimerization of the Met complexes, signals increased cell migration, proliferation, and metastasis through intracellular activation of tyrosine-kinase phosphorylation circuits.^2^ Indeed, the Met receptor has been a target of cancer therapy. In the present study, we selected the Met receptor associated with the MCF-7 cell membrane as a functional interface to integrate a synthetic DNA network on the cell membrane for the dynamic, transient, network-guided dimerization of the Met receptors and downstream cell motility functions. The system and its mode of operation are displayed in Figure 3A. Four nucleic acid components, E_1_/T_1_, E/Q_1_, F_1_/T_1_, and F/Q_1_, are integrated into the cell membrane. Each of the nucleic acid units E, E_1_, F, and F_1_ includes an anti-Met aptamer domain that allows the anchoring and binding of the respective components to the Met receptor associated with the cell membrane. The nucleic acids E_1_ and F_1_ are caged with T_1_ to form components E_1_/T_1_ and F_1_/T_1_, whereas units E and F are caged by a short strand Q_1_. In addition, components F_1_ and F are modified with Cy5 and Cy3 fluorophores, respectively, providing a reliable tool for probing the dynamic feature of the circuits (*vide infra*). The engineered caged structures of the components prohibit any reconfiguration of the components in the “Rest” framework integrated into the membrane. In the presence of the auxiliary nickase, Nt.BbvCI, and the fuel strand T_1_′, the triggered reconfiguration of the components into a dynamic CDN “N” proceeds, thus leading to the CDN-guided, spatially proximate aptamer-induced dimerization of the Met receptors, while the transient downstream cell migration function proceeds. The fuel strand T_1_′ uncages components E_1_/T_1_, F_1_/T_1_, to form the duplex T_1_/T_1_′, while the released strands E_1_ and F_1_ displace the caging strands Q_1_, leading to a dynamically emerged CDN “N” composed of the constituents EE_1_, EF_1_, FE_1_, and FF_1_. As each of the components comprising the constituents includes the Met aptamer, the emergence of the CDN is anticipated to costabilize the Met-dimer formation. That is, the interbridged duplex constituents allosterically stabilize the Met-dimer formation in CDN “N”. Dimerization of the Met receptors provides then the downstream signaling for the functional migration of the cells. Moreover, as the fuel strand T_1_′ is pre-engineered to allow the nicking of the T_1_/T_1_′ duplex by the nickase, the release of T_1_ displaces the equilibrated constituents associated with the membrane, leading to the recovery of “Rest” components that are diffusionally separated within the cell membrane. Separation of the Met-dimer complexes blocks the signaling path, leading to transient dynamic inhibition of the cell motility functions. Nevertheless, refueling the framework with T_1_′ reactivates the dynamic transient signaling of migration/motility functions. For the “in solution” T_1_′-triggered transition of E_1_/T_1_, E/Q_1_, F_1_/T_1_, and F/Q_1_ (lacking the conjugated Met protein) into CDN “N” and the nickase-induced transient recovery of the “Rest” components, Figure S22 and accompanying discussion.

**Figure 3:**
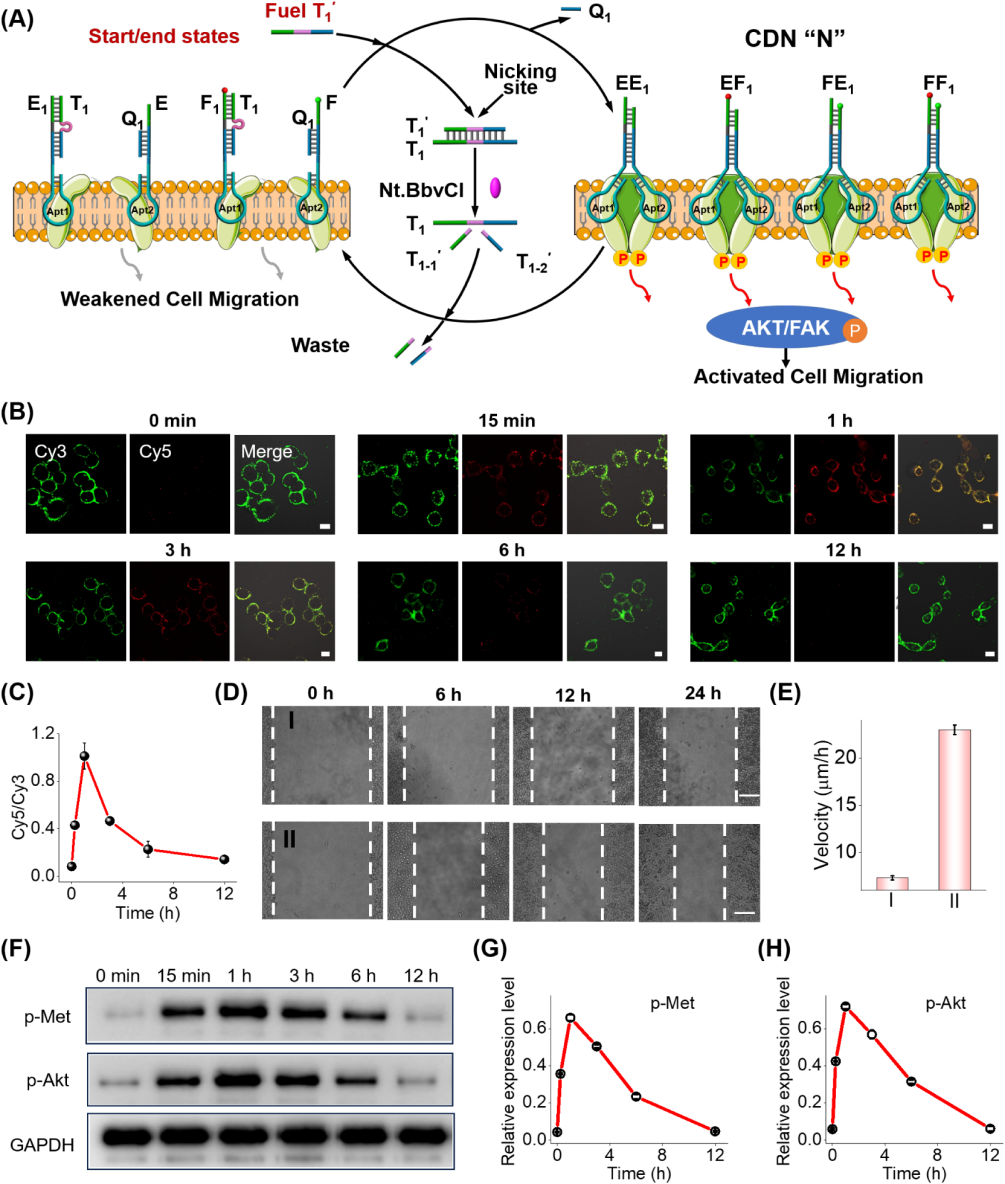
Fuel-driven reconfiguration of a cell membrane-anchored reaction module into a dynamic network signaling the transient cellular AKT/FAK network guiding cell migration. (A) Schematic integration of four Met-aptamer-modified components in MCF-7 cell membranes and their T_1_′-triggered, transient reconfiguration into a CDN signaling the transient intracellular Akt/FAK phosphorylation pathway and activating cell migration. (B) Temporal confocal microscopy images probing the transient formation and depletion of the CDN in the cell membrane by the FRET signal of Cy3/Cy5 fluorophores associated with the respective CDN constituents. (For a high-resolution image of the cells, see Figure S23.) Scale bar: 20 μm. (C) Transient integrated Cy5/Cy3 FRET intensities corresponding to the T_1_′-fueled formation of the CDN “N” and the concomitant nickase-stimulated depletion of the CDN “N”. (D) Temporal images corresponding to the wound healing assay probing the rates of cell mobility of a scratched domain separating two populations of cells containing the Met-aptamer-modified components: panel I-Upper run, in the absence of T_1_′-fueled reconfiguration of the CDN “N”; panel II-Lower run, subjected to the T_1_′/nickase-triggered formation/depletion of CDN “N”. Scale bar: 200 μm. (E) Healing rates of the scratched domain by migration of cells functionalized with the four Met-aptamer-modified components in the absence of fuel T_1_′ (i), and subjected to the T_1_′/nickase-triggered formation and depletion of CDN “N” (ii) derived from Figure 3D. (F) Western blots corresponding to the time-dependent analysis of phosphorylated Met (p-Met) and phosphorylated Akt (p-Akt) within the course of transient formation and depletion of CDN “N”. GAPDH is employed as an internal control standard that does not participate in the dynamic signaling network. Transient temporal expression levels of p-Met (G) and p-Akt (H) are shown with the course of the dynamic formation/depletion of CDN “N”. Results are derived from (F).

The T_1_′-triggered dynamic transition of the parent reaction circuit into CDN “N” composed of the duplex-stabilized Met-dimer constituents and the transient depletion of CDN “N” to the parent circuit were demonstrated by confocal fluorescence imaging following the dynamic process proceeding in the cell membrane at different time intervals, Figure 3B. As stated, strand F is labeled with fluorophore Cy3, while strand F_1_ is labeled with Cy5. The triggered formation of the duplex constituent FF_1_ in CDN “N” includes the fluorophores Cy3/Cy5 in spatial proximity, allowing a FRET process between Cy3 and Cy5. That is, upon excitation of Cy3, energy transfer to Cy5 is anticipated to induce the fluorescence of Cy5. Thus, the FRET signal and its transient depletion within the cell membrane can be probed at time intervals of operation of the dynamic evolution of CDN “N”. Figure 3B shows the confocal fluorescence microscopy images probing at different time intervals of the dynamic process, the fluorescence features of the cells through the Cy3 channel and Cy5 channel upon exciting Cy3. At *t* = 0, only the fluorescence of Cy3 (green color) is observed, consistent with the lack of any FRET process. After 15 min and along a time interval of 1 h, the FRET signal is evidenced by the decreased Cy3 and intensified Cy5 fluorescence (red). After this time interval, the FRET signal is gradually depleted, and after 12 h only the green fluorescence of Cy3 is observed. These results are consistent with the dynamic reconfiguration features of the network displayed in Figure 3A. Within the first 1 h of applied trigger T_1_′, CDN “N” is evolved in the cell membrane and afterward the nickase-driven separation of CDN “N” leads within 12 h to the transient separation of CDN “N” and the recovery of the parent reaction circuit. By analyzing, at each time interval, different frames and probing the integrated FRET intensities of Cy3/Cy5, the dynamic pattern of the FRET intensities was evaluated, Figure 3C. A transient evolution of CDN “N” within a time interval of 1 h is observed, followed by a slower transient depletion of the FRET signal. It should be noted that this dynamic fluorescence patterns could be recycled by reapplying the trigger T_1_′ on the circuit-modified cells, implying that the Met-aptamer reaction circuit stays intact within the cell membrane along the dynamic reconfiguration process. (For further experiments addressing the stability and dynamic recyclability of the transient operation of CDN “N” on the MCF-7 cell membrane, and the stability of the parent reaction module on the MCF-7 cell membrane in the culture media, see Figure S24 and accompanying discussion.) Moreover, the potential cytotoxicity of CDN “N” associated with the MCF-7 cell was examined, Figure S21B. No effect on the cell viability was observed.

The CDN “N”-controlled dissipative signaling of the MCF-7 cells is reflected in the macroscopic cell migration functions. Figure 3D depicts the cell migration assay probing the rates of migrative bridging of a scratched domain separating two populations of the MCF-7 cells functionalized with the “Rest” reaction module. In entry I, the cells are not activated by the trigger T_1_′, whereas in entry II, the identical system was subjected to the trigger T_1_′. Evidently, the migrative occupation of the gap by the T_1_′-triggered CDN “N”-loaded cells (entry II) is substantially faster than the migrative occupation of the gap by the nontriggered cells (entry I). Figure 3E presents quantitatively the rates of migrative gapping of the scratched domain by the CDN “N” signaling Met-dimer-induced migration of the cells, (i), in comparison to the nontriggered reference consisting of the “Rest” framework-loaded cells, (ii). Evidently, the rates of migrative occupation of the scratched domain, (i), reveal faster migration than cells without treatment, (ii). Figure 3F presents the Western blotting of p-Met, p-Akt, and GAPDH as an internal reference in MCF-7 cell lysates, at different time intervals of the transient CDN-guided intracellular phosphorylation pathway. Figure 3G and 3H depicts the transient expression yields of p-Met and p-Akt. Within the first 1 h, the contents of p-Met and p-Akt are intensified in the cell lysates, consistent with the CDN-guided stabilization of the Met-dimer signaling the downstream intracellular phosphorylation pathways. At longer time intervals, the contents of p-Met and p-Akt gradually decrease, and after a time interval of 12 h, their contents in the cell lysates are depleted. These results are consistent with the transient CDN “N”-guided dimerization of Met controlling the signaling of the intracellular phosphorylation pathway. While the buildup of CDN “N” stabilized the Met-dimer and the accompanying signaling of intracellular phosphorylation, the temporal transient depletion of CDN “N” separated the Met-dimer structures accompanied by the blockage of the phosphorylation processes. (For further control experiments supporting the transient CDN “N” guided signaling of the p-Akt pathway, see Figure S25 and accompanying discussions.)

Finally, the transient and switchable dynamic reconfiguration of CDN “N” within the cell membrane and the associated signaling of the cellular motility were probed by following, at different time intervals, the cell migration trajectories and their statistical analysis. As shown in Figure 4A and Movies S1–S4, Panel I exemplifies the typical single-cell migration trajectories within the course of the T_1_′-triggered dynamic cycle evolving CDN “N”, followed by the nickase-stimulated separation of CDN “N” into a nonsignaled cell configuration revealing inhibited motility. The motility trajectories of single-cell samples, observed within separated time intervals of 2 h, in the absence of an applied trigger, are displayed in (i) and (ii). Evidently, the cells have low migration distances. After these time intervals, the cells are subjected to the trigger T_1_′ and the motility distances of single cells are further examined within two successive, 2 h, time intervals. Evidently, subjecting the cells to trigger T_1_′ results in an obvious increase in the migration distances of single-cell samples (iii) and (iv). These results are consistent with the temporal T_1_′-induced evolution of CDN “N” within the cell membrane, signaling cell motility functions. Subsequently to these two time intervals demonstrating a temporal increase in the migration trajectories, the migration trajectories of single-cell samples with two successive time intervals are displayed in (v) and (vi), respectively. Within the next time interval of 2 h, a clear decrease in the distance presented in the migration trajectory is observed, and in the last 2 h time interval, the migration trajectory is dampened to the pattern of the cell prior to the application of the trigger T_1_′. These results are consistent with the nickase-stimulated transient separation of CDN “N” and the recovery of the inactive separated reaction module in the cell membrane. Figures 4A, Panels II–III depict a collective analysis of the trajectories of ten different cells operating within the time interval of 12 h. The temporal migration distances demonstrate a transient pattern revealing an initial increase in the migration distances, followed by a temporal decrease in the migration distances. Similarly, the rates of motility of the cells in Figure 4A, Panel III reveals an initial increase followed by a decrease in the motility rates. After a time interval of 12 h, the migration rates are blocked. Moreover, subjecting the cells to a second T_1_′-triggered cycle reactivated the formation of the active CDN “N” within the cell membrane, resulting in the transient signaling and accompanying migration/motility functions of the cells, Figure 4B. The cyclic operation of the transient motility of the cells indicates that the circuit and CDN retain a stable configuration within the cell membrane.

**Figure 4:**
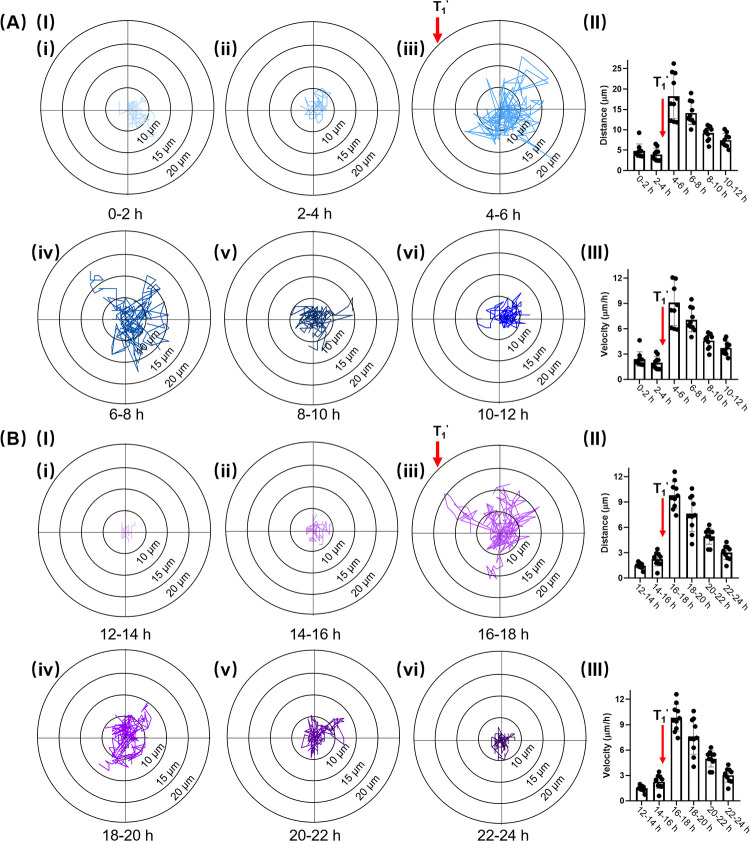
Fuel-driven transient reconfigured cell membrane-anchored network signaling transient single-cell migration trajectories. (A) Panel I-Time-dependent migration trajectories of single cells induced by the T_1_′-triggered transient CDN “N” signaling on the MCF-7 cell membrane. Panel II-Transient distances executed at different time intervals by a collection of ten different MCF-7 cells, driven by the transient signaling of CDN “N” associated with the cell membranes. Panel III-Transient velocities of migration associated with a collection of nine MCF-7 cells driven at different time intervals by the T_1_′-triggered transient operation of CDN “N” associated with the cells. (B) Second T_1_′-triggered activation of CDN “N” stimulated a transient migration cycle of the cells: Panel I Time-dependent trajectories of single cells. Transient temporal distances (Panel II) and transient temporal migration velocities (Panel III) at time intervals executed by a collection of nine cells.

## Conclusions

The field of Systems Chemistry is advanced by introducing a new versatile concept whereby a nucleic acid circuit is integrated with cell membranes, yielding a functional hybrid framework. Auxiliary triggers reconfigure the membrane-embedded circuit into a CDN signaling system that temporally controls intracellular functions. Specifically, we demonstrated the anchoring of four pre-engineered DNA components that include the Met-aptamer sequence within MCF-7 cell membranes. In the presence of an auxiliary trigger T_1_′ and a coupled nicking enzyme (Nt.BbvCI), the triggered reconfiguration of the circuit into a transiently, operating CDN composed of four constituents allosterically stabilizing spatially proximate Met-dimer complexes within the cell membranes, was demonstrated. The four constituents in the CDN include encoded compositional information to be separated into the parent circuit, in which the Met receptor dimers are separated. The temporal-network-guided formation of the Met-dimer signals the intracellular Akt-phosphorylation pathway, activating macroscopic cell migration/motility functions. As a result, the synthetic DNA circuit driving dynamic, transient control of the allosterically stabilized Met-dimer formation/depletion was implemented for guiding temporal migration/motility functions of the cell. The major accomplishments of the present studies are reflected by the successful integration of stable DNA dynamic networks in cell membranes, allowing cyclic, switchable, and transient signaling of cell migration functionalities. It should be noted that the allosterically aptamer-driven dimerization of the Met receptors could be achieved by a pair of aptamer constituents,^55^ rather than the use of a four-constituent dynamic CDN network used in our study. Nevertheless, the use of four constituent dynamic network (CDN) has important advantages: (i) The four-constituent dynamic framework provides a programmable stability-controlled allosteric affinity binding of the target protein that might be perturbed by the incorrect orientation of an aptamer dimer.^61^ Indeed, these advantages of a CDN framework over an allosteric dimer pair have been recently addressed for improved sensing. (ii) The availability of four constituents in the signaling CDN could allow the engineering of two orthogonal pairs carrying aptamers for two different types of receptors embedded in the cell membrane. This would allow, in the future, the orthogonal signaling of different cell functionalities (by using CDN frameworks of higher dimensionality,^62^ the complexity of signaling could be even further enhanced).

The development of dynamic synthetic network/cell hybrid platforms included several steps: (i) The engineering and characterization of the nucleic acid circuits that include the appropriate functionalities of recognition units, reconfiguration functionalities, and transducing elements probing the dynamic features of the systems. (ii) The integration of the Met-aptamer-modified circuit into cell membranes and characterization of the dynamic, transient reconfiguration of the circuit within the cell membrane into a temporal CDN. (iii) Demonstration that the formation/depletion of the temporally operating CDN leads to network-guided transient formation of Met-dimer complexes within the cell membrane signaling the intracellular phosphorylation pathway controlling transient migration/motility functions of the cells.

Beyond introducing an arsenal of experimental tools to probe and characterize the dynamic features of the DNA network within the cell membrane, the significant novelty of the system is reflected by the versatile possibilities that broaden this concept. For example, many other cell membrane receptors, such as PTK7, VEGFR1, or HER2, may be used to anchor functional DNA circuits into the cell membrane for subsequent triggered dynamic reconfiguration into network-guided cell signaling pathways. Also, other auxiliary triggers, such as switchable aptamer-ligand complexes, dynamic DNA triplexes, or light stimuli, could be implemented to activate the temporal signaling pathways. Moreover, control over other cell functionalities, e.g., biocatalytic networks and cell apoptosis, could be of immense therapeutic impact. Albeit, our results demonstrated the stability of the hybrid DNA circuits/cell systems, the stability of the frameworks in biological environments might be a future challenge to resolve. Nevertheless, the use of modified thiophosphate oligonucleotides or locked nucleic acid structures could overcome stability issues.^63,64^

## Supplementary Material











## Notes

The detailed design and operation of transient CDNs, cell experiments, characterization of cell imaging and migration including confocal microscopy measurements, flow cytometry analysis, and cell wound healing analysis, the simulation modules and constant rates of the dynamic transient DNA circuits and evolution of CDN, additional results supporting the main text (PDF)
(MP4)
(MP4)
(MP4)
(MP4)
