# Supplementary material for: Cell-Membrane-Anchored Synthetic Dynamic DNA Circuits for Signaling Transient Cell Migration
Source: J Am Chem Soc. 2025 Sep 12;147(38):34292–302. doi: 10.1021/jacs.5c03070 (PMC12464974; doi:10.1021/jacs.5c03070)
Supplement: Supplementary file 1 [file ja5c03070_si_001.pdf]

Supporting Information

Cell-Membrane-Anchored Synthetic Dynamic DNA Circuits for Signaling Transient Cell Migration

Nina Lin[1‡], Yu Ouyang[2‡], Yunlong Qin[2], Songqin Liu[1], Itamar Willner[2*], Yuanjian Zhang, Zhixin Zhou[1*]

[1] School of Chemistry and Chemical Engineering, Southeast University, Nanjing 211189, China.

[2] Institute of Chemistry, The Hebrew University of Jerusalem, Jerusalem 91904, Israel.

*Corresponding author. Email: willnea@vms.huji.ac.il; zhixin.zhou@seu.edu.cn

‡ These authors contributed equally.

Materials and Methods

Materials. Tris-Acetate-EDTA (TAE) buffer solution, DNA Marker A (25-500 bp), and "GelRed nucleic acid gel stain" were purchased from Sangon Biotech Co., Ltd. (Shanghai, China). Gel Fast Preparation Kits for polyacrylamide gel electrophoresis (PAGE) were purchased from Shanghai Epizyme Biomedical Technology Co., Ltd (China). Nicking enzyme, Nt.BbvCI, (10,000 units mL^{-1}, 2.3 μM), EcoRI, BamHI, and rCutSmart™ Buffer (10 ×, pH = 7.9) were purchased from New England BioLabs Inc.. Human Embryonic Kidney 293 Cells (HEK-293T) and corresponding culture medium were purchased from Wuhan Servicebio Technology Co., Ltd. (China). MCF-7 Cells, DMEM culture medium, and Fetal Bovine Serum were purchased from KeyGEN BioTECH (China). Trypsin-EDTA (0.25%) and phosphate buffered saline (1×, pH = 7.4) were purchased from KeyGEN BioTECH (China). Ultrapure water (resistance > 18 MΩ·cm) was used in all of the experiments.

All DNA oligonucleotides were synthesized by Sangon Biotech Co., Ltd. The oligonucleic acid sequences used in the study include (from 5' end to 3' end):

A: GATATCAGCGATACAGAAGAACCCCCATCACAAA

A-Cy3: GATATCAGC-Cy3-GATACAGAAGAACCCCCATCACAAA

A_1: GGTTTGTGATGGACGTTCTTCTGTCACCCATGTTACTCT

B: CCATTCAGCGATACAGAAGAACCCCCATCACAAA

B-Cy3: CCATTCAGC-Cy3-GATACAGAAGAACCCCCATCACAAA

B_1: GGTTTGTGATGGACGTTCTTCTGTCACCCATGTTTCAGT

T_1: GACAGAAGAACGCTGAGGCCATCACAAACC

T_1': GATGGCCTCAGCGTT

T_1-Cy3: Cy3-GACAGAAGAACGCTGAGGCCATCACAAACC

T_1'-BHQ2: GATGGCCTCAGCGTT-BHQ2

Q: TCTTCTGTATC-BHQ2

C: GATATCAGC-Cy3-GATACAGAAGAACCCCCATCACAAATTT-cholesterol

C_1: cholesterol-TTTGGTTTGTGATGGACGTTCTTCTGTCACCCATGTTACTCT

D: CCATTCAGC-Cy5-GATACAGAAGAACCCCCATCACAAATTT-cholesterol

D_1: cholesterol-TTTGGTTTGTGATGGACGTTCTTCTGTCACCCATGTTTCAGT

E: GATACAGAAGAACCCCCATCACAAATTT*ATCAGGCTGGATGGTAGCTCGGTCGGGG TGGGTGGGTTGGCAAGTCTGAT*

F(Cy3): Cy3-GATACAGAAGAACCCCCATCACAAATTT*ATCAGGCTGGATGG TAGCTCGGTCGGGGTGGGTGGGTTGGCAAGTCTGAT*

E_1: *CGTGTCACGGATGGTAGCTCGGTCGGGGTGGGTGGGTTGGCAGTGACACG*TTTGGT TTGTGATGGACGTTCTTCTGT*C*

F_1(Cy5): *CGTGTCACGGATGGTAGCTCGGTCGGGGTGGGTGGGTTGGCAGTGACACG*TTT GGTTTGTGATGGACGTTCTTCTGTC-Cy5

Q_1: TCTTCTGTATC

For gel:

A-gel: TTTTTTTGATATCAGCGATACAGAAGAACCCCCATCACAAA

A_1-gel: GGTTTGTGATGGACGTTCTTCTGTC

Substrate sequences for DNAzyme subunits associated with consitituents:

Sub 1 (AA_1/CC_1): ROX-AGAGTATrAGGATATC-BHQ2

Sub 2 (BB_1/DD_1): ROX-ACTGAATrAGGAATGG-BHQ2

Sub 3 (BA_1/DC_1): FAM-AGAGTATrAGGAATGG-BHQ1

Sub 4 (AB_1/CD_1): ROX-ACTGAATrAGGATATC-BHQ2

The respective DNAzyme subunit sequences in each of oligonucleic acid are underlined. The sequences for recognition of Met receptor are italic.

Measurements. Fluorescence spectra and time-dependent fluorescence changes were recorded with a Cary Eclipse Fluorometer (Agilent Technologies). The excitations of fluorophores FAM, Cy3, ROX, and Cy5 were performed at 496, 546, 588, and 646 nm, respectively, while the emissions of FAM, Cy3, ROX, and Cy5 were recorded at 516, 566, 608, and 666 nm, respectively. The fluorescence spectra of Cy3 were collected from 556 to 750 nm with excitation wavelength at 546 nm. UV/vis spectra were performed on a Cary 60 UV/Vis spectrometer (Agilent Technologies). The PAGE were run on VE-180 electrophoresis units (Tanon, China). The fluorescence changes of dissipative system on cell membrane were monitored with Zeiss confocal laser scanning microscope (LSM 900, Japan), and all images were analyzed with image J. The FRET signal of Cy5 on the cell membrane was collected with emission signal ranging from 645 to 700 nm and using an external 561 nm excitation, while the external 640 nm excitation with an emission signal collection ranging from 645 to 700 nm was selected for the fluorophore Cy5. The fluorescence of Cy3 on the cell membrane was collected with emission signal ranging from 4 to 620 nm and using an external 561 nm excitation

Dissipative evolution of DNA-based constitutional dynamic networks (CDNs). For dissipative evolution of a [2×2] CDN "K" shown in Fig. 1A, the DNA strands, A, B, A_1, B_1, 1 μM each, T_1, 2.2 μM, and Q, 2,4 μM, were mixed in 1 × rCutSmart™ Buffer containing 20 mM tris-acetate, 50 mM potassium acetate, 10 mM magnesium acetate, 100 μg/ml recombinant albumin (pH 7.9@25°C). The mixture was annealed at 40 °C for 5 min, and then cool to 25 °C at a rate of 0.2 °C min^{-1}, followed by incubation at 25 °C for 60 min. The as-prepared mixture (150 μL) was subjected to the different concentrations of nicking enzyme, Nt.BbvCI, and then the resulting mixture was transferred into the cuvette. To activate the dissipative evolution of CDN "K", different concentrations of fuel T_1' were added to the mixture, and time-dependent fluorescence changes were followed spectroscopically at 37 °C.

For the modification of the HEK-293T cells with the cholesterol-functionalized circuits shown in Figure 2, the following procedure was implemented. The HEK-293T cells were cultured in a DMEM medium that included 10% FBS. The cells were grown till an 80% cover-density of the culture was obtained. The cells detached from the culture into a PBS buffer solution were purified by repeated buffer rinsing/precipitation cycles, and then subjected to the cholesterol-functionalized constituents. The duplexed DNA strands, cholesterol-C/Q, cholesterol-D/Q, cholesterol-C_1/T_1, and cholesterol-D_1/T_1, 12 μM each, were incubated with HEK-293T cells (*ca.* 1.5×10^5) for 30 min. The DNA-immobilized HEK-293T cells were washed with culture medium twice to remove excessive DNA components, and then were resuspended in 200 μL culture medium. For optimization of loading of the circuits on the cell membrane, see Figure S11. The resulting round-shaped, living HEK-293T cells were characterized by confocal fluorescence microscopy imaging. For fluorescence and bright-field images of the HEK-293T cells after treatment with the constituent circuits, see Figure S12B. For related reference addressing the detachment of the cells from the culture, their modification with cholesterol-modified nucleic acids, and the round-shape morphology of the cholesterol-nucleic acid-modified cells, see reference (1)-(3).

Reference:

1. You, M.; Lyu, Y.; Han, D.; Qiu, L.; Liu, Q.; Chen, T.; Sam Wu, C.; Peng, L.; Zhang, L.; Bao, G.; Tan, W., DNA probes for monitoring dynamic and transient molecular encounters on live cell membranes. Nat. Nanotechnol. 2017, 12, 453.

2. Yang, S.; Wang, M.; Tian, D.; Zhang, X.; Cui, K.; Lü, S.; Wang, H.-h.; Long, M.; Nie, Z., DNA-functionalized artificial mechanoreceptor for de novo force-responsive signaling. Nat. Chem. Biol. 2024, 20, 1066.

3. Xiao, M.; Lai, W.; Yu, H.; Yu, Z.; Li, L.; Fan, C.; Pei, H., Assembly Pathway Selection with DNA Reaction Circuits for Programming Multiple Cell–Cell Interactions. J. Am. Chem. Soc. 2021, 143, 3448.

Subsequently, different concentrations of nicking enzyme, Nt.BbvCI, and 1 × rCutSmart™ Buffer were added to the culture medium. To activate the dissipative evolution of CDN on cell membrane, different concentrations of fuel T_1' were added to the mixture, and fluorescence intensity on the membrane surface at different time intervals was recorded by confocal laser scanning microscope, and the fluorescent images were analyzed by ImageJ software.

For operation of dissipative evolution on MCF-7 cell membrane shown in Figure 3, the MCF-7 cells were seeded in confocal dish for 24 h. Then, the duplexed DNA strands, E/Q_1, F/Q_1, E_1/T_1, and F_1/T_1, 0.5 μM each, were incubated with MCF-7 cells for 20 min at 37 °C. The DNA-immobilized MCF-7 cells were washed with culture medium twice to remove excessive DNA components. Subsequently, different concentrations of nicking enzyme, Nt.BbvCI, and 1 × rCutSmart™ Buffer were added to the culture medium. To activate the dissipative evolution of CDN on cell membrane, different concentrations of fuel T_1' were added to the mixture, and fluorescence intensity on the membrane surface at different time intervals was recorded by confocal laser scanning microscope, and the fluorescent images were analyzed by ImageJ software.

Cell culture. HEK-293T cells were grown Basal medium supplemented with 10% Fetal Bovine Serum and 1% Penicillin-Streptomycin Solution in a cell culture incubator at 37 °C with 5 % CO_2. MCF-7 cells were cultured in DMEM with 10% Fetal Bovine Serum and 1% Penicillin-Streptomycin Solution in a cell culture incubator at 37 °C with 5 % CO_2.

Confocal microscopy measurements. 100 μL of suspended DNA-immobilized HEK-293T cells prepared as described above, was added to a glass bottom cell culture dish 35 mm in diameter (NEST, China). Upon the addition of fuel T_1', the fluorescence intensity was monitored during the dissipative cycle. At least 20 cells were recorded at each time interval. Image analysis was performed with ImageJ software. To record the recyclable property of the dissipative system, the

average fluorescence intensity of Cy3 and Cy5 on the membrane surface was measured by ImageJ software.

Flow cytometry analysis. The fluorescence intensity of Cy3 and Cy5 on HEK-293T cells along the dissipative cycle was analyzed by flow cytometry using a BD FACSCalibur (FACS101) system operated at a mid-pressure. The fluorescence intensity of HEK-293T cells was detected by following λ_{ex} and λ_{em}: λ_{ex} = 488 nm laser, λ_{em} = 564 - 606 nm for Cy3 and λ_{ex} = 488 nm laser, λ_{em} > 650 nm for Cy5. All FCS data were determined for a total of 10,000 events. Data were analyzed with FlowJo 10.8.1 software.

Cell Wound Healing Analysis. The MCF-7 cells were cultured in a 6-well plate that was divided into two groups. After the cell density reached 90%, the cells in each well were scratched with a 200 µL pipet tip. Each well was washed three times with PBS buffer to remove the detached cells. Group 1 was selected as a control without treatment in DMEM and 1 × rCutSmart™ Buffer. Group 2 was incubated with parent reaction module for 20 min and washed two times with PBS buffer. Subsequently, the nicking enzyme, Nt.BbvCI, and fuel T_1' were added to the Group 2 to activate the dissipative process in DMEM and 1 × rCutSmart™ Buffer. The width of the wound was recorded at different time intervals by a microscope (10 × objective).

Cell viability assay. HEK-293T and MCF-7 cells were seeded into 96-well plate at the density of ca. 1×10^4 cells per well and incubated overnight. Then, cells subjected to the DNA circuits at different time-intervals. The cells were washed three times with PBS to remove the excessive circuits. Subsequently, cell viability was quantified using the CCK-8 assay. 10 µL of CCK-8 solution and 100uL of DEME was added into each well. Cells were incubated for approximately 30 minutes at 37°C and 5% CO_2. Finally, the absorbance at 450 nm (OD 450) of the wells was measured with a microplate reader (Thermo Scientific).

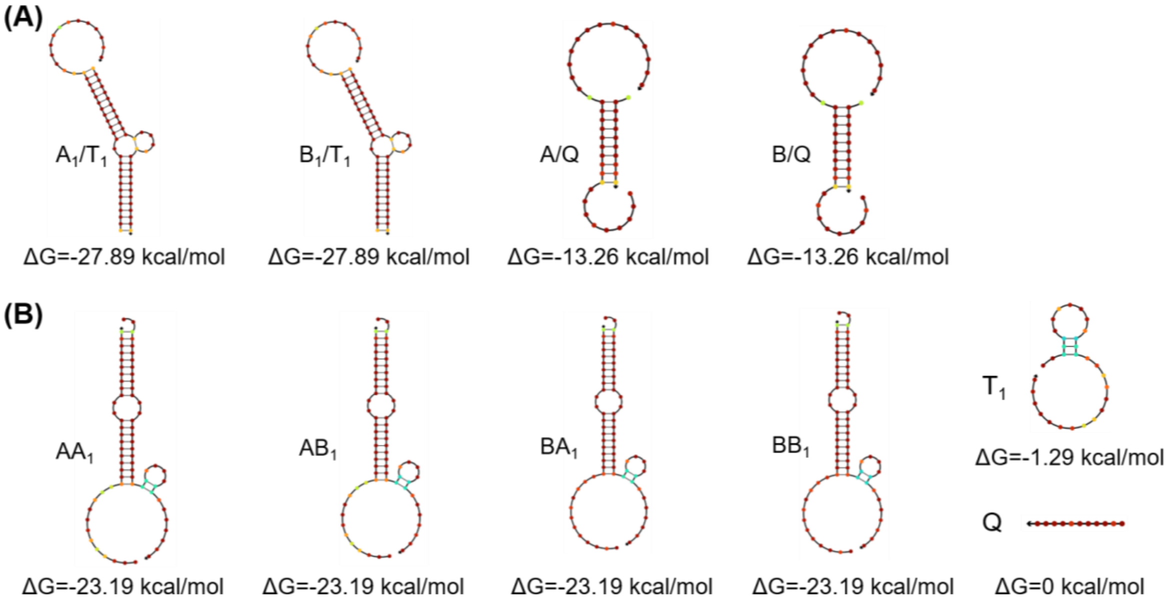

Figure S1. The Gibbs energy balance associated with the transient formation and depletion of CDN "K" shown in Figure 1A. The predicted secondary structure and Gibbs energies of parent state (A) and intermediates (B).

$$(1)\ A_1/T_1 + B_1/T_1 + A/Q + B/Q \underset{\Delta G_2}{\overset{\Delta G_1}{\rightleftharpoons}} \frac{1}{2}AA_1 + \frac{1}{2}AB_1 + \frac{1}{2}BA_1 + \frac{1}{2}BB_1 + 2T_1 + 2Q$$

$$(2)\ \Delta G_1 = -82.30\ \text{kcal/mol}$$

$$(3)\ \Delta G_2 = -48.96\ \text{kcal/mol}$$

$$(4)\ \Delta G_1 < \Delta G_2$$

Figure S1A depicts the stabilized configuration of these constituents associated with the "rest" reaction module and the Gibbs free energies of the constituents following the base sequences of the respective components. Figure S1B depicts the stabilized configuration of the constituents of CDN "K" and the Gibbs free energies associated with the different constituents. (Note that the base-sequence of constituents include single strand bi-loop non-paired domains that do not shown in the schematic structure of CDN "K" to facilitate the recovery of CDN "K" to the parent state.) The Gibbs free energy associated with the parent reaction module corresponds to ΔG_1 = -82.30 kcal mol^{-1}, whereas the Gibbs free energy associated with the intermediate CDN "K" is ΔG_2 = -48.96 kcal mol^{-1}. This implies that $\Delta G_1 < \Delta G_2$, supporting the energy-driven transition of CDN "K" to the parent module.

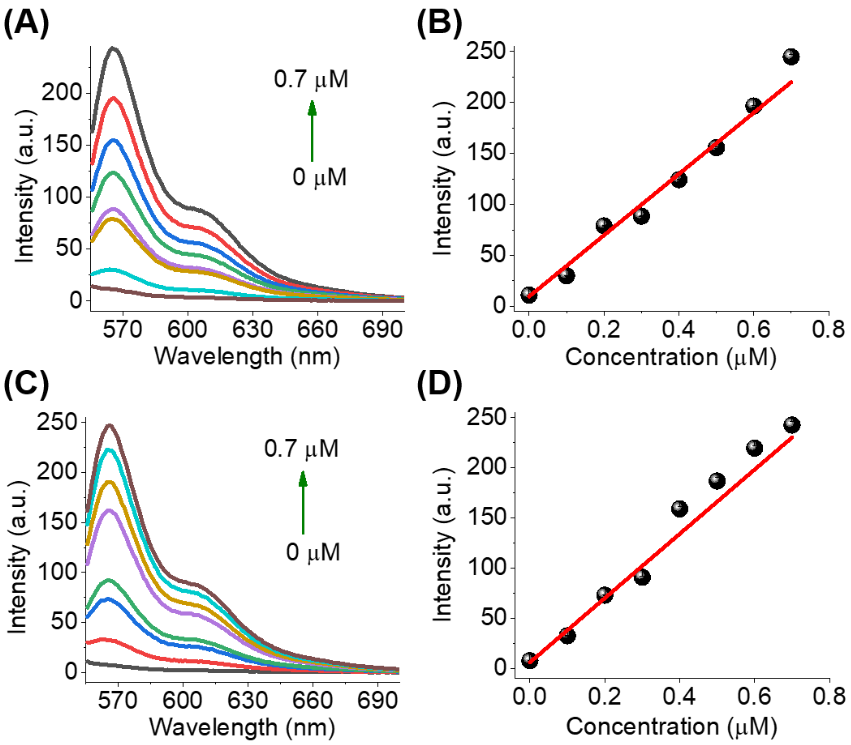

Figure S2. Calibration curves of the fluorescence changes of Cy3 at different concentrations of (A_1+B_1). (A) Fluorescence spectra of duplex A/Q in the presence of different concentrations of (A_1+B_1). (B) The derived calibration curve corresponding to the fluorescence changes of Cy3-labeled A as a function of concentrations of (A_1+B_1). (C) Fluorescence spectra of duplex B/Q upon the addition of different concentrations of (A_1+B_1). (D) The derived calibration curve corresponding to the fluorescence changes of Cy3-labeled B as a function of different concentrations of (A_1+B_1). Results are presented as mean ± standard deviation (n = 3).

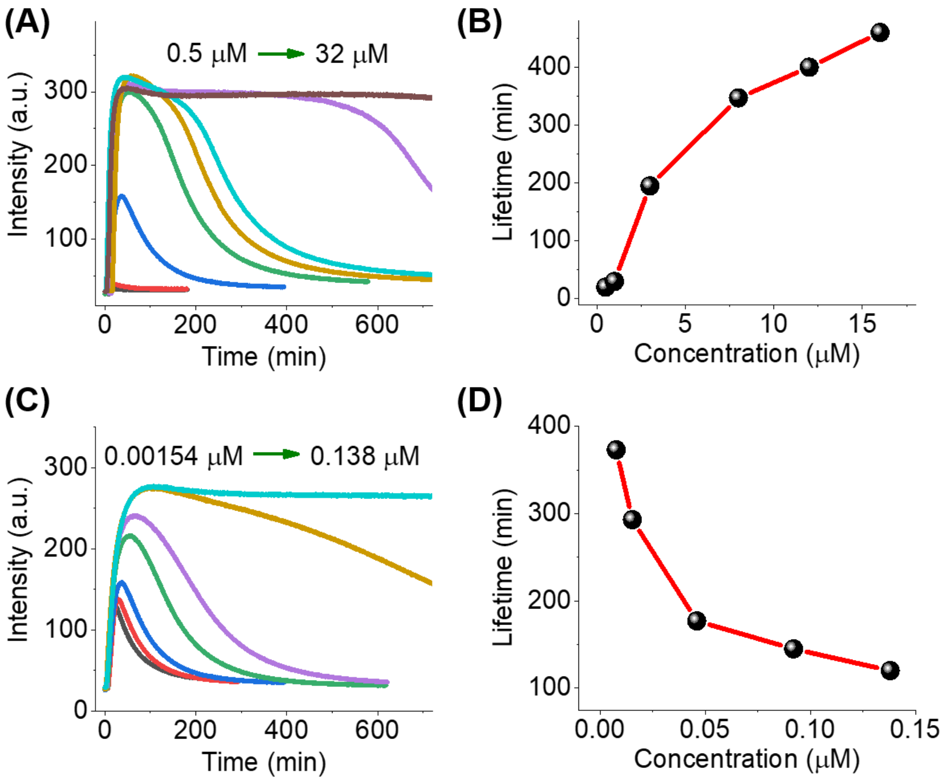

Figure S3. (A) Transients corresponding to the dissipative network in the presence of different concentrations of the trigger T_1'. (B) Plots corresponding to the respective transient lifetime at different concentrations of T_1'. (C) Transients corresponding to the dissipative network in the presence of different concentrations of the nicking enzyme. (D) Plots corresponding to the respective transient lifetime at different concentrations of nicking enzyme.

Computational simulation of the time-dependent concentrations of the constituents in transient CDN "K"

To computationally simulate the time-dependent concentration changes of the constituents in CDN "K" upon T_1'-triggered transient formation and depletion of CDN "K", we formulated a kinetic model that comprises the stepwise reactions associated with the dynamic T_1'-triggered transition of parent modules to CDN "K" and the nickase-guided recovery of CDN "K" to the parent module. The sub-reactions are summarized in eq. (1) ~ eq. (14), where eq. (1) ~ eq. (4) present the assembly of the parent module, eq. (5) ~ eq. (11) depicts the T_1'-triggered formation of the CDN "K" comprising of AA_1, AB_1, BA_1, BB_1, and eq. (12) ~ eq. (14) represents the nickase-guided cleavage of the fuel strand, leading to the depletion of CDN "K" and recovery of parent module.

The rate equations corresponding to the respective sub-reactions were defined as equations, Figure S4. These rate equations were coded in the Matlab R2019b software while providing k_i/k_{-i} values of the set of rate equations, together with the initial concentrations of the constituents in the system at t = 0 min (the k_i/k_{-i} values were based on literature values of related duplexes and provide approximate inputs), resulting in the first dynamic curves corresponding to the temporal concentrations of the constituents. As the experimental temporal concentrations of the constituents are read as a data matrix, the nonlinear least-squares solver (Lsqcurvefit) embedded in Matlab software compares the first round of simulated data experimental data and initiates an optimization iteration under the optimization limits: ′StepTolerance′,1e-90, ′FunctionTolerance′,1e-15, ′OptimalityTolerance′, 1e-20, ′MaxFunctionEvaluation′, 10000). This simulation procedure is processed till a satisfactory fit between the simulated results and experimental results is obtained. Usually, the maximum number of 500 stimulated iterations yield a satisfactory fit between the computationally simulated results and experimental data leading to a set of rate-constants that are supposed to follow the kinetic model. To support the computationally simulated results, it is well desirable to compare one (or more) experimentally validated rate constant to the simulated values. Alternatively, to avoid a possible meaningless set of computationally derived rate constants, the significance of the computational results may be supported by predicting the temporal concentrations of the constituents at different auxiliary conditions and validation of the predicted values by experiments.

Kinetic equations for dissipative evolution system shown in Figure 1A:

$$(1)\ A+Q \underset{k_{-1}}{\overset{k_1}{\rightleftarrows}} AQ$$

$$(2)\ B+Q \underset{k_{-2}}{\overset{k_2}{\rightleftarrows}} BQ$$

$$(3)\ A_1+T_1 \underset{k_{-3}}{\overset{k_3}{\rightleftarrows}} A_1T_1$$

$$(4)\ B_1+T_1 \underset{k_{-4}}{\overset{k_4}{\rightleftarrows}} B_1T_1$$

$$(5)\ A_1T_1+T_1' \underset{k_{-5}}{\overset{k_5}{\rightleftarrows}} A_1+T_1T_1'$$

$$(6)\ B_1T_1+T_1' \underset{k_{-6}}{\overset{k_6}{\rightleftarrows}} B_1+T_1T_1'$$

$$(7)\ A_1+AQ \underset{k_{-7}}{\overset{k_7}{\rightleftarrows}} Q+AA_1$$

$$(8)\ A_1+BQ \underset{k_{-8}}{\overset{k_8}{\rightleftarrows}} Q+BA_1$$

$$(9)\ B_1+AQ \underset{k_{-9}}{\overset{k_9}{\rightleftarrows}} Q+AB_1$$

$$(10)\ B_1+BQ \underset{k_{-10}}{\overset{k_{10}}{\rightleftarrows}} Q+BB_1$$

$$(11)\ AA_1+BB_1 \underset{k_{-11}}{\overset{k_{11}}{\rightleftarrows}} BA_1+AB_1$$

$$(12)\ T_1T_1'+E \underset{k_{-12}}{\overset{k_{12}}{\rightleftarrows}} T_1T_1'E$$

$$(13)\ T_1T_1'E \xrightarrow{k_{13}} E+T_1T_{1-1}{}'T_{1-2}{}'$$

$$(14)\ T_1T_{1-1}{}'T_{1-2}{}' \underset{k_{-14}}{\overset{k_{14}}{\rightleftarrows}} T_1+T_{1-1}{}'+T_{1-2}{}'$$

Derivatives:

$$\frac{dA}{dt}=k_{-1}[AQ]-k_1[A][Q]$$

$$\frac{dQ}{dt}=k_{-1}[AQ]-k_1[A][Q]+k_{-2}[BQ]-k_2[B][Q]+k_7[A_1][AQ]-k_{-7}[Q][AA_1]+k_8[A_1][BQ]-k_{-8}[Q][BA_1]+$$
$$k_9[B_1][AQ]-k_{-9}[Q][AB_1]+k_{10}[B_1][BQ]-k_{-10}[Q][BB_1]$$

$$\frac{dAQ}{dt}=k_1[A][Q]-k_{-1}[AQ]+k_{-7}[Q][AA_1]-k_7[A_1][AQ]+k_{-9}[Q][AB_1]-k_9[B_1][AQ]$$

$$\frac{dB}{dt}=k_{-2}[BQ]-k_2[B][Q]$$

$$\frac{dBQ}{dt}=k_2[B][Q]-k_{-2}[BQ]+k_{-8}[Q][BA_1]-k_8[A_1][BQ]+k_{-10}[Q][BB_1]-k_{10}[B_1][BQ]$$

$$\frac{dA_1}{dt}=k_{-3}[A_1T_1]-k_3[A_1][T_1]+k_5[A_1T_1][T_1']-k_{-5}[A_1][T_1T_1']+k_{-7}[Q][AA_1]-k_7[A_1][AQ]+k_{-8}[Q][BA_1]-k_8[A_1][BQ]$$

$$\frac{dT_1}{dt}=k_{-3}[A_1T_1]-k_3[A_1][T_1]+k_{-4}[B_1T_1]-k_4[B_1][T_1]+k_{14}[T_1T_{1-1}'T_{1-2}']-k_{-14}[T_1][T_{1-1}'][T_{1-2}']$$

$$\frac{dA_1T_1}{dt}=k_3[A_1][T_1]-k_{-3}[A_1T_1]+k_{-5}[A_1][T_1T_1']-k_5[A_1T_1][T_1']$$

$$\frac{dB_1}{dt}=k_{-4}[B_1T_1]-k_4[B_1][T_1]+k_6[B_1T_1][T_1']-k_{-6}[B_1][T_1T_1']+k_{-9}[Q][AB_1]-k_9[B_1][AQ]+k_{-10}[Q][BB_1]-k_{10}[B_1][BQ]$$

$$\frac{dB_1T_1}{dt}=k_4[B_1][T_1]-k_{-4}[B_1T_1]+k_{-6}[B_1][T_1T_1']-k_6[B_1T_1][T_1']$$

$$\frac{dT_1'}{dt}=k_{-5}[A_1][T_1T_1']-k_5[A_1T_1][T_1']+k_{-6}[B_1][T_1T_1']-k_6[B_1T_1][T_1']$$

$$\frac{dT_1T_1'}{dt}=k_5[A_1T_1][T_1']-k_{-5}[A_1][T_1T_1']+k_6[B_1T_1][T_1']-k_{-6}[B_1][T_1T_1']+k_{-12}[T_1T_1'\mathrm{E}]-k_{12}[T_1T_1'][E]$$

$$\frac{dAA_1}{dt}=k_7[A_1][AQ]-k_{-7}[Q][AA_1]+k_{-11}[BA_1][AB_1]-k_{11}[AA_1][BB_1]$$

$$\frac{dBA_1}{dt}=k_8[A_1][BQ]-k_{-8}[Q][BA_1]+k_{11}[AA_1][BB_1]-k_{-11}[BA_1][AB_1]$$

$$\frac{dAB_1}{dt}=k_9[B_1][AQ]-k_{-9}[Q][AB_1]+k_{11}[AA_1][BB_1]-k_{-11}[BA_1][AB_1]$$

$$\frac{dBB_1}{dt}=k_{10}[B_1][BQ]-k_{-10}[Q][BB_1]+k_{-11}[BA_1][AB_1]-k_{11}[AA_1][BB_1]$$

$$\frac{dE}{dt}=k_{-12}[T_1T_1'\mathrm{E}]-k_{12}[T_1T_1'][E]+k_{13}[T_1T_1'E]$$

$$\frac{dT_1T_1'E}{dt}=k_{12}[T_1T_1'][E]-k_{-12}[T_1T_1'\mathrm{E}]-k_{13}[T_1T_1'E]$$

$$\frac{dT_1T_{1-1}'T_{1-2}'}{dt}=k_{13}[T_1T_1'E]+k_{-14}[T_1][T_{1-1}'][T_{1-2}']-k_{14}[T_1T_{1-1}'T_{1-2}']$$

$$\frac{dT_{1-1}'}{dt}=k_{14}[T_1T_{1-1}'T_{1-2}']-k_{-14}[T_1][T_{1-1}'][T_{1-2}']$$

$$\frac{dT_{1-2}'}{dt}=k_{14}[T_1T_{1-1}'T_{1-2}']-k_{-14}[T_1][T_{1-1}'][T_{1-2}']$$

Figure S4. Simulated model for dissipative evolution of CDN “K” shown in Figure 1A. The kinetic scheme involved in the dissipative system was built based on above equations. Knowing the time-dependent concentration changes of the constituents, along the dissipative evolution of CDN “K”, we computationally simulated the time-dependent concentration changes of constituents by using Matlab R2019b. The computationally simulated rate constants are summarized in Table S1.

Table S1. Rate constants derived from the computational simulation of the dissipative FRET system shown in Figure 1.

k_1	67 $\mu M^{-1}\cdot min^{-1}$	k_{-5}	8.44 $\mu M^{-1}\cdot min^{-1}$	k_{10}	0.065 $\mu M^{-1}\cdot min^{-1}$
k_{-1}	3.11 min^{-1}	k_6	1.8 $\mu M^{-1}\cdot min^{-1}$	k_{-10}	0.007 $\mu M^{-1}\cdot min^{-1}$
k_2	55 $\mu M^{-1}\cdot min^{-1}$	k_{-6}	6.5 $\mu M^{-1}\cdot min^{-1}$	k_{11}	0.16 $\mu M^{-1}\cdot min^{-1}$
k_{-2}	5.61 min^{-1}	k_7	0.082 $\mu M^{-1}\cdot min^{-1}$	k_{-11}	0.15 $\mu M^{-1}\cdot min^{-1}$
k_3	18.5 $\mu M^{-1}\cdot min^{-1}$	k_{-7}	0.0065 $\mu M^{-1}\cdot min^{-1}$	k_{12}	11.2 $\mu M^{-1}\cdot min^{-1}$
k_{-3}	8×10^{-5} min^{-1}	k_8	0.085 $\mu M^{-1}\cdot min^{-1}$	k_{-12}	0.7 min^{-1}
k_4	15.6 $\mu M^{-1}\cdot min^{-1}$	k_{-8}	0.0058 $\mu M^{-1}\cdot min^{-1}$	k_{13}	$0.7\cdot min^{-1}$
k_{-4}	1×10^{-4} min^{-1}	k_9	0.08 $\mu M^{-1}\cdot min^{-1}$	k_{14}	9.2 min^{-1}
k_5	1.72 $\mu M^{-1}\cdot min^{-1}$	k_{-9}	0.0063 $\mu M^{-1}\cdot min^{-1}$	k_{-14}	0.04 $\mu M^{-2}\cdot min^{-1}$

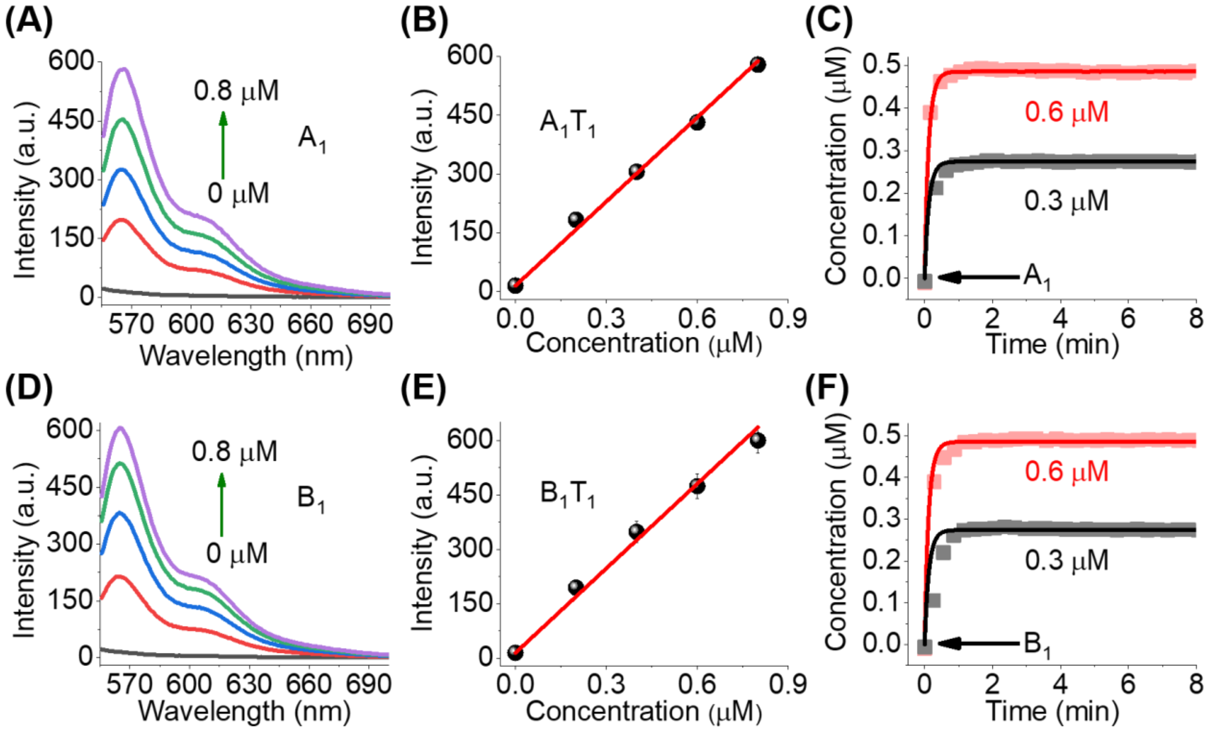

Figure S5. Determination of the rate constants, k_5/k_{-5} and k_6/k_{-6}, in kinetic equations shown in Figure S4. (A) The fluorescence spectra of fluorophore/quencher-modified T_1/T_1' in response to different concentrations of A_1. (B) The fluorescence intensity T_1/T_1' as a function of concentration of A_1. (C) Time-dependent concentration changes of T_1/T_1' upon the addition of different concentrations of A_1. (D) The fluorescence spectra of fluorophore/quencher-modified T_1/T_1' upon the addition of different concentrations of B_1. (E) The fluorescence intensity T_1/T_1' as a function of concentration of B_1. (F) Time-dependent concentration changes of T_1/T_1' upon the addition of different concentrations of B_1. Results are presented as mean ± standard deviation (n = 3).

To experimentally determine the rate constants, k_5/k_{-5}, in kinetic equations shown in Figure S4, the fluorophore/quencher-modified T_1/T_1' was subjected to two different concentrations of A_1 (0.3 μM and 0.6 μM), and the time-dependent concentration changes of T_1/A_1 were evaluated by following time-dependent fluorescence changes of Cy3-labeled T_1, and transducing them into contents by using the calibration curve shown in Figure S5A and S5B. From the kinetic profiles shown in Figure S5C and using the Matlab R2019b program, the respective $k_5 = 1.72$ μM^{-1} min^{-1} and $k_{-5} = 8.44$ μM^{-1} min^{-1} was derived. As shown in Figure S5D-5F, the respective rate constants, $k_6 = 1.8$ μM^{-1} min^{-1} and $k_{-6} = 6.5$ μM^{-1} min^{-1} were also derived.

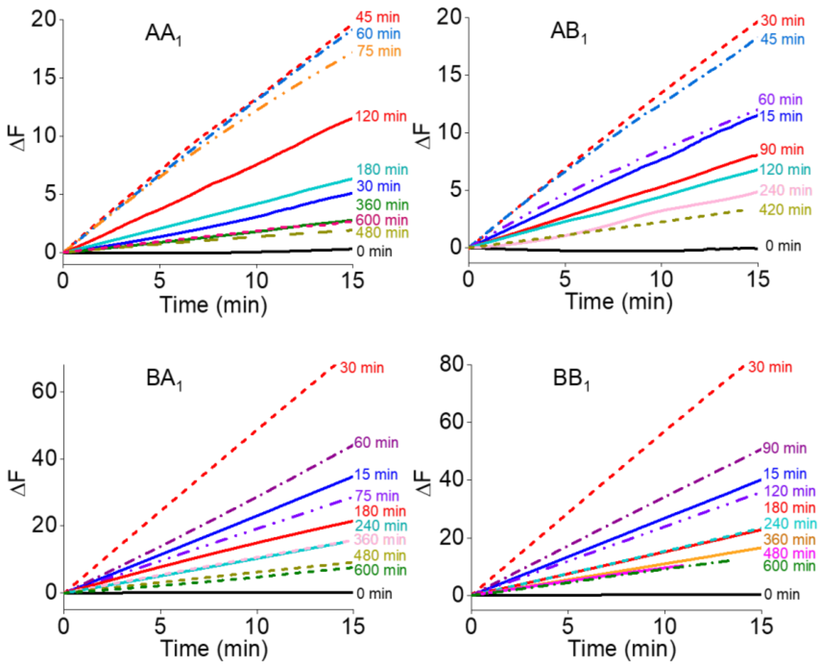

Figure S6. Time-dependent fluorescence changes generated by DNAzymes associated with the constituents in CDN "K" at different time intervals upon subjecting the "Rest" reaction module to T_1', 3 μM, in the presence of nickase, 0.069 μM.

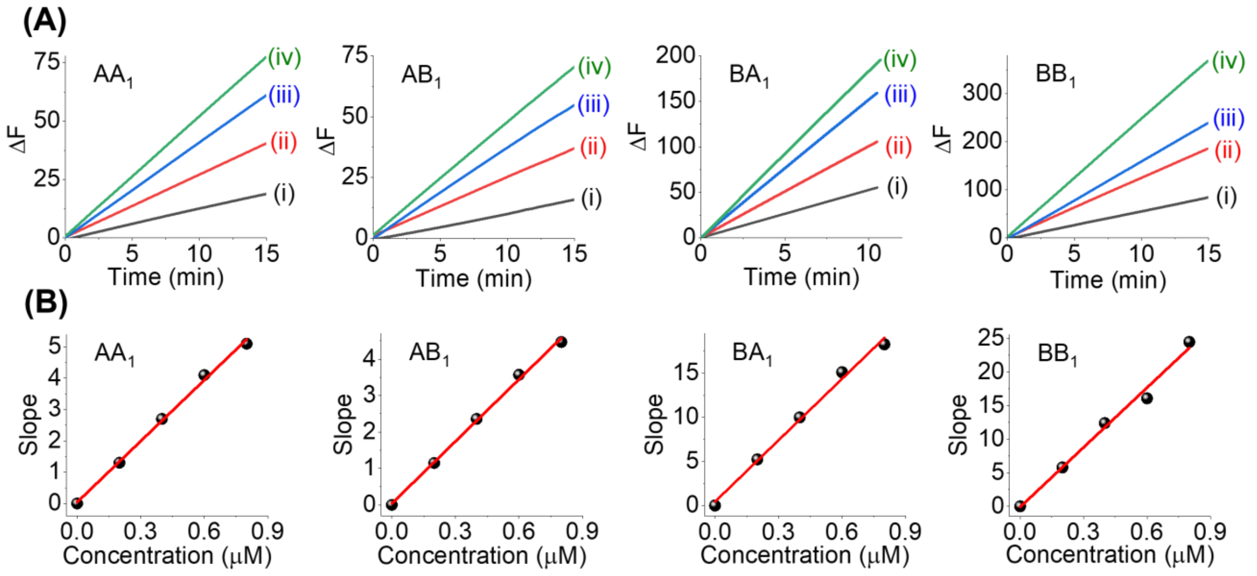

Figure S7. Calibration curves of the rates of cleavage of the fluorophore/quencher-functionalized substrates by different concentrations of DNAzyme associated with constituents in CDN "K". (A) Time-dependent fluorescence changes generated upon the cleavage of the fluorophore/quencher-modified substrates by the respective DNAzyme reporter units associated with the individual intact constituents at variable concentrations: (i) 0.2 μM, (ii) 0.4 μM, (iii) 0.6 μM, and (iv) 0.8 μM. (B) The derived calibration curves of the catalytic rates of the DNAzymes associated with respective constituents as a function of their concentrations.

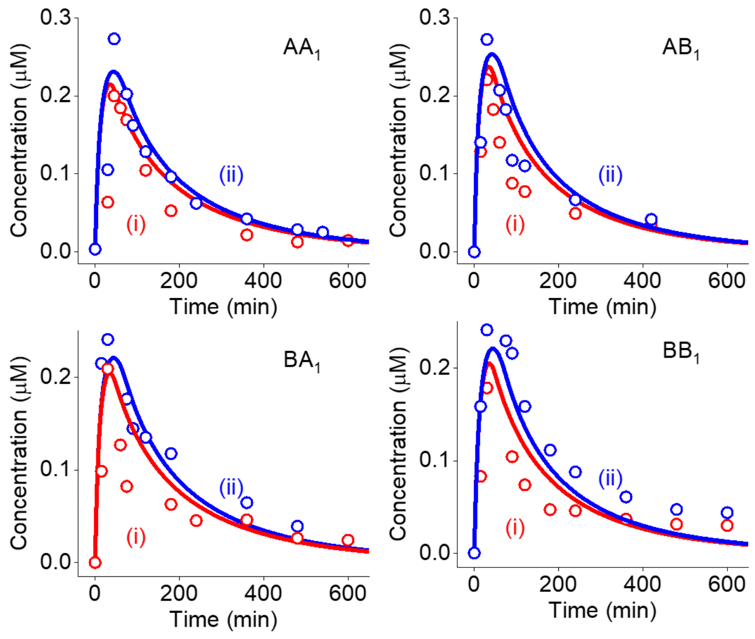

Figure S8. The effect of concentration of nicking enzyme on the dissipative evolution of CDN "K". Time-dependent concentration changes of DNAzyme units associated with the constituents during T_1'-fueled dissipative evolution of CDN "K" at a fixed concentration of fuel T_1', 3 μM, and in the presence of different concentrations of nicking enzyme: (i) 0.069 μM and (ii) 0.046 μM.

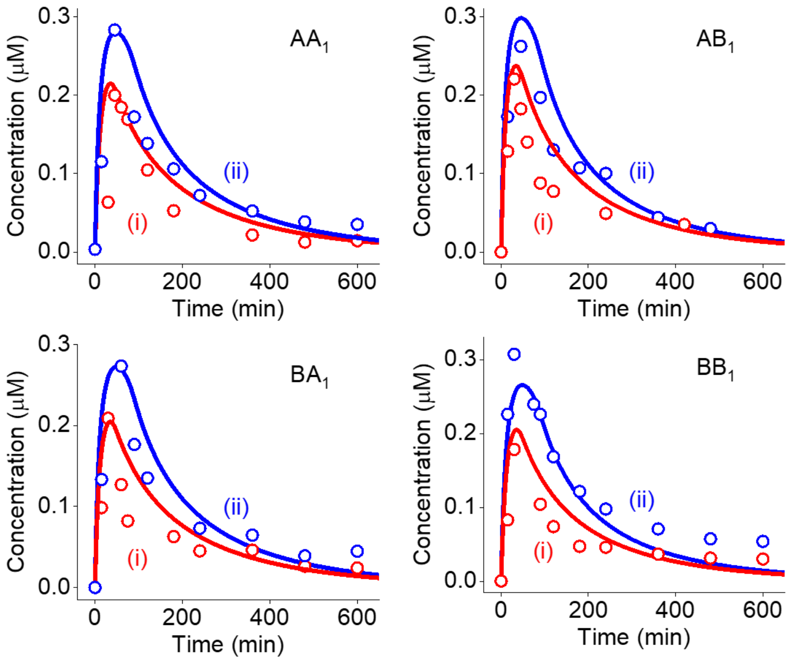

Figure S9. The effect of concentrations of fuel T_1' on the dissipative evolution of CDN "K". Time-dependent concentration changes of DNAzymes units associated with the constituents during T_1'-fueled dissipative evolution of CDN "K" at a fixed concentration of nicking enzyme, 0.069 μM, and in the presence of different concentrations of fuel T_1': (i) 3 μM and (ii) 5 μM.

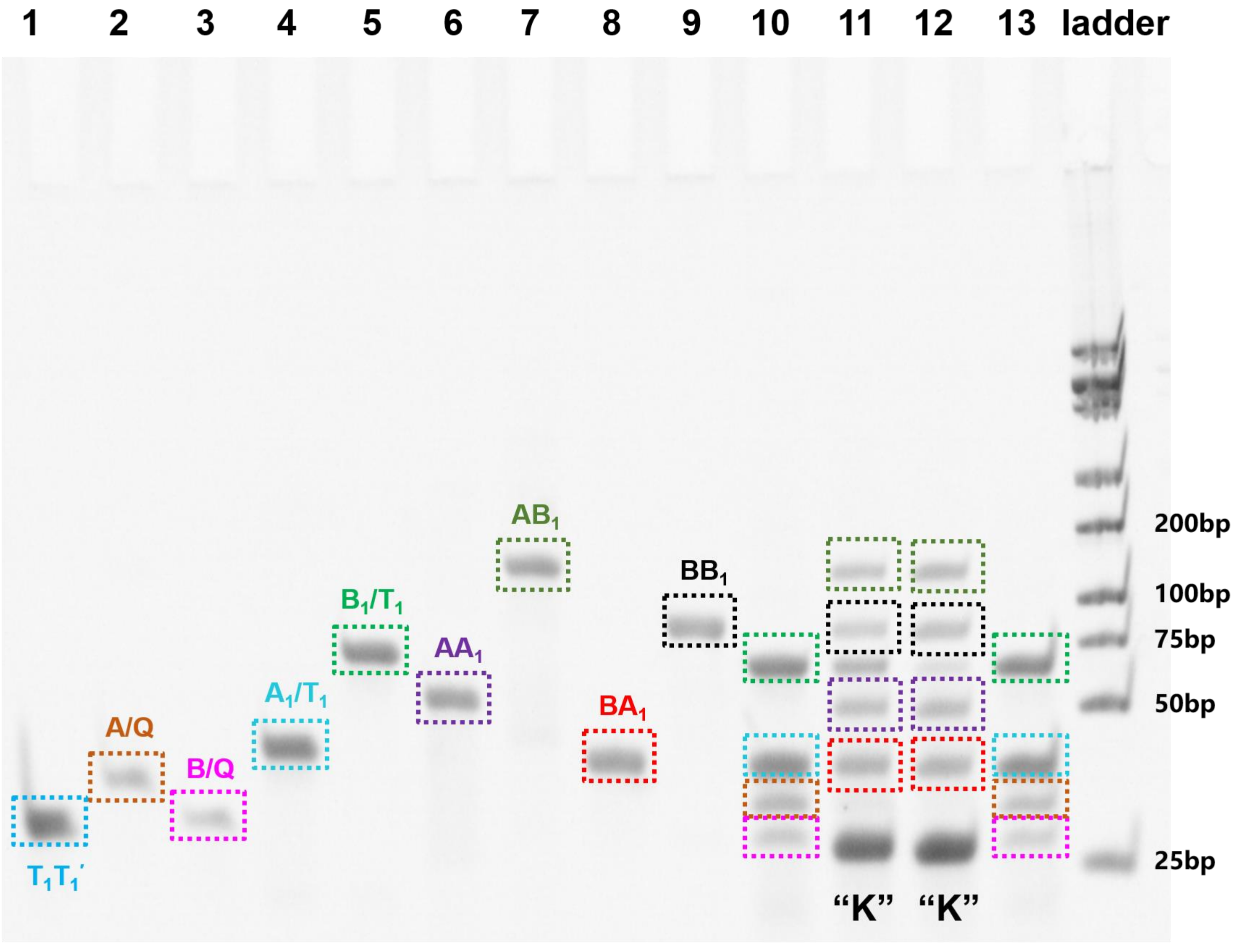

Figure S10. Gel electrophoresis (native) demonstrating T_1'-fueled dissipative evolution of CDN "K". Lane 1 - T_1T_1'; lane 2 - A/Q; lane 3 - B/Q; lane 4 - A_1/T_1; lane 5 - B_1/T_1; lane 6 - AA_1; lane 7 - AB_1; lane 8 - BA_1; lane 9 - BB_1; lane 10 - the original system before subjecting the system to T_1'; lane 11 corresponding to the separated constituents associated with CDN "K" upon the addition of T_1' to the dissipative system for a time interval of 30 min; lane 12 - the addition of T_1' to the system in the absence of nicking enzyme; lane 13 - corresponding to the separated constituents after a time interval of 12 h following the addition of T_1' to the dissipative system.

For comparison, the intact bands of the duplex strands, T_1T_1' (lane 1), A/Q (lane 2), B/Q (lane 3), A_1/T_1 (lane 4), and B_1/T_1 (lane 5), and individual intact constituents AA_1 (lane 6), AB_1 (lane 7), BA_1 (lane 8), and BB_1 (lane 9) are provided. After subjecting the dissipative system to the T_1' for a time interval of 30 min, the separated constituents comprising CDN "K" are clearly observed, as shown in lane 11, while the bands corresponding to the separated constituents were disappeared and the system restored to the original state after subjecting the dissipative system to the T_1' for a time interval of 12 h (lane 13). Thus, the results clearly demonstrated the fuel T_1' could guide the dissipative evolution of CDN "K".

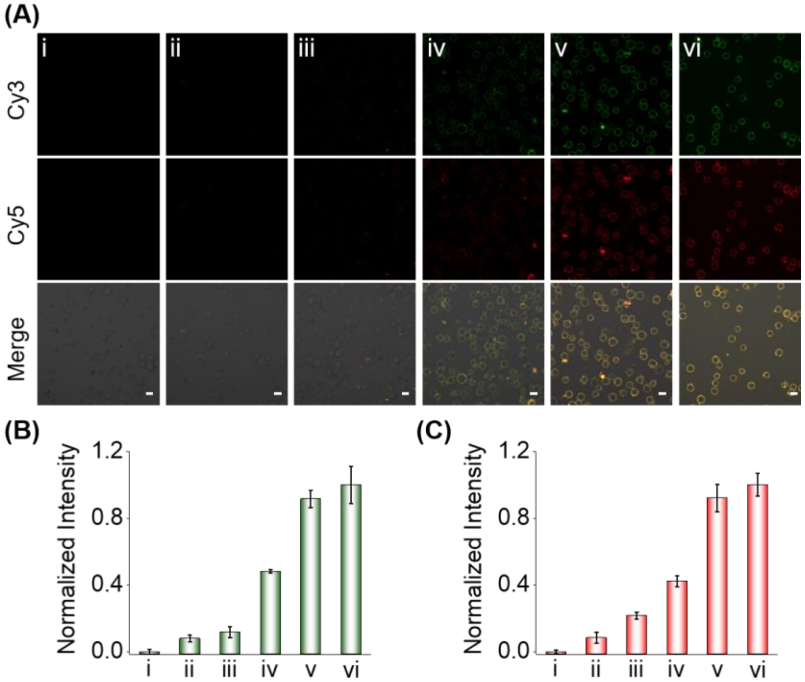

Figure S11. (A) Temporal confocal microscopy images of HEK-293T cells subjected to the cholesterol-modified DNA strands with different concentrations: (i) 0.3 μM, (ii) 0.6 μM, (iii) 1 μM, (iv) 3 μM, (v) 6 μM, and (vi) 12 μM. The derived integrated confocal fluorescence intensities of Cy3 (B) and Cy5 (C) from Figure S11A.

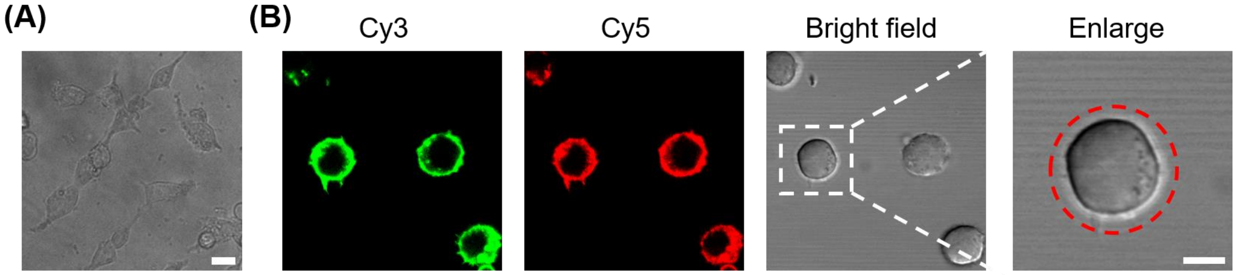

Figure S12. Confocal (bright-field or fluorescence) images corresponding to (A) adherent state of HEK-293T cells, and (B) the round-shape non-adherent HEK-293T cells treated with the cholesterol-modified constituents.

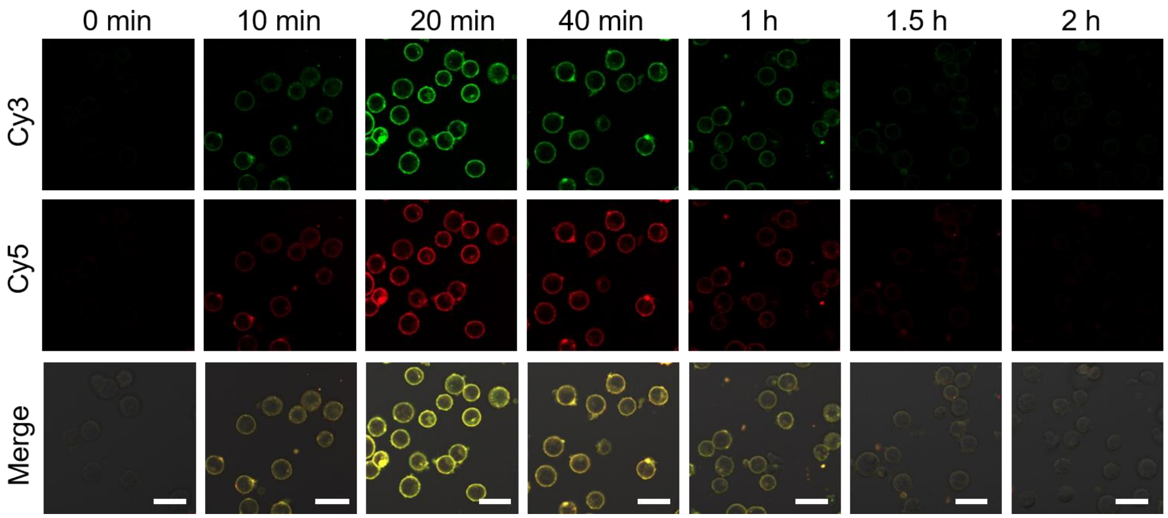

Figure S13. Temporal confocal fluorescence microscopy images corresponding to fluorophore Cy3 (green) associated with the constituents (CC_1+CD_1), fluorophore Cy5 (red) associated with the constituents (DC_1+DD_1), and bright-field overlay (yellow), upon the T_1'-triggered evolution/depletion of CDN "M", in the presence of T_1' = 30 nM. Scale bar, 20 μm.

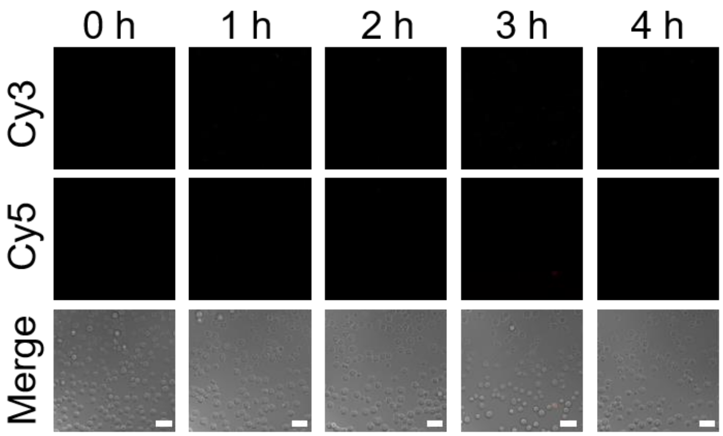

Figure S14. Fluorescence microscopy images of dissipative networks on cell membrane in the absence of fuel T_1' yet at a fixed concentration of nicking enzyme. Scale bar is 20 μm.

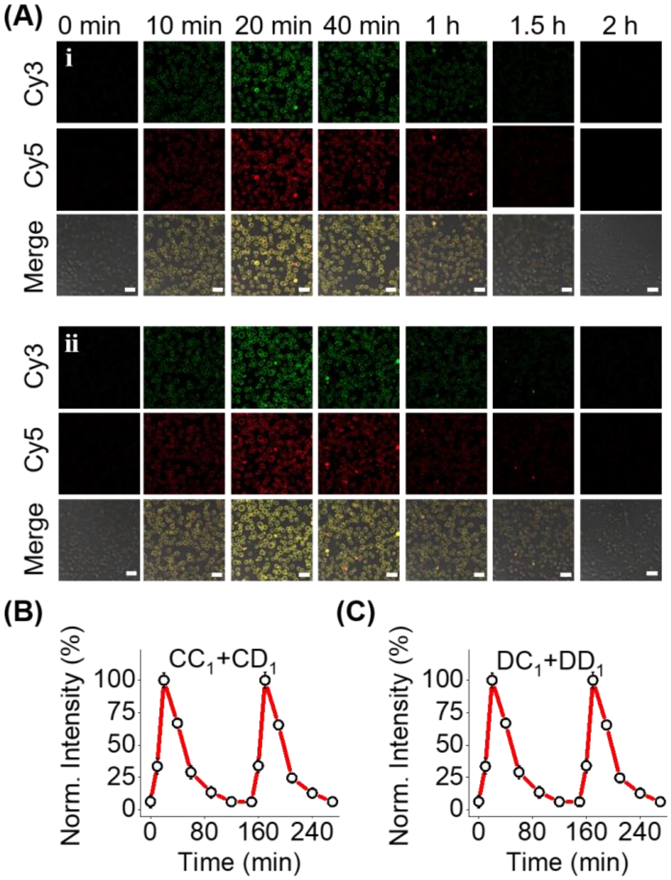

Figure S15. Operation of transient lifecycles of CDN "M" on the cell membrane. Operation of two transient lifecycles on the cell membrane, upon readdition of the fuel strand T_1', prior to each cycle. Scale bar, 20 μm.

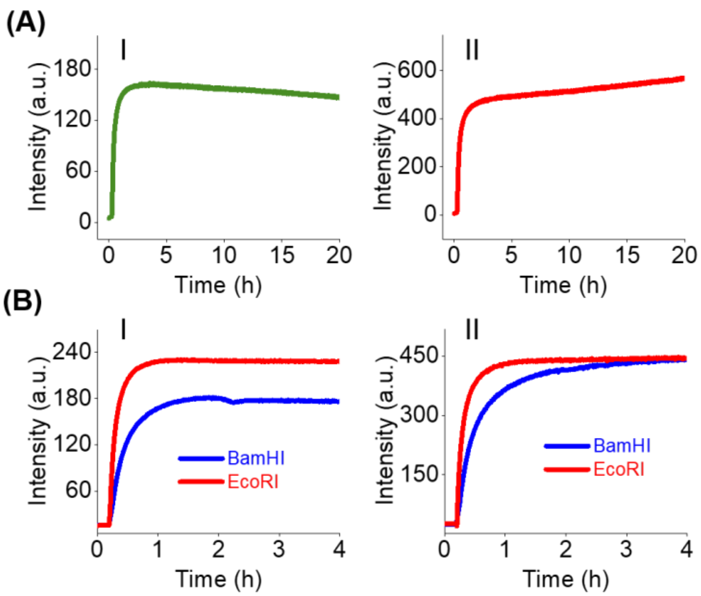

Figure S16. (A) Time-dependent fluorescence changes of the constituents (CC_1+CD_1), (I), and (DC_1+DD_1), (II), upon the T_1'-triggered formation of CDN "M", in the presence of the fuel strand T_1' yet in the absence of nicking enzyme. (B) Time-dependent fluorescence changes of the constituents (CC_1+CD_1), (I), and (DC_1+DD_1), (II), upon the T_1'-triggered formation of CDN "M", in the presence of the fuel strand T_1' and different enzymes.

Figure S16 depicts the significance of the nickase on the transient dynamic recovery of CDN "M" to the parent module, and the specificity of nicakse on this dynamic transformation. In the absence of nicakse (Nt.BbvCI), no fluorescence changes (depletion) of constituents (CC_1+CD_1) and (DC_1+DD_1) is observed, Figure S16A, Panels I and II. Similarly, Figure S16B, Panels I and II, demonstrates that the nicking enzyme BamHI or the endonuclease EcoRI have no effect on fluorescence changes (depletion).

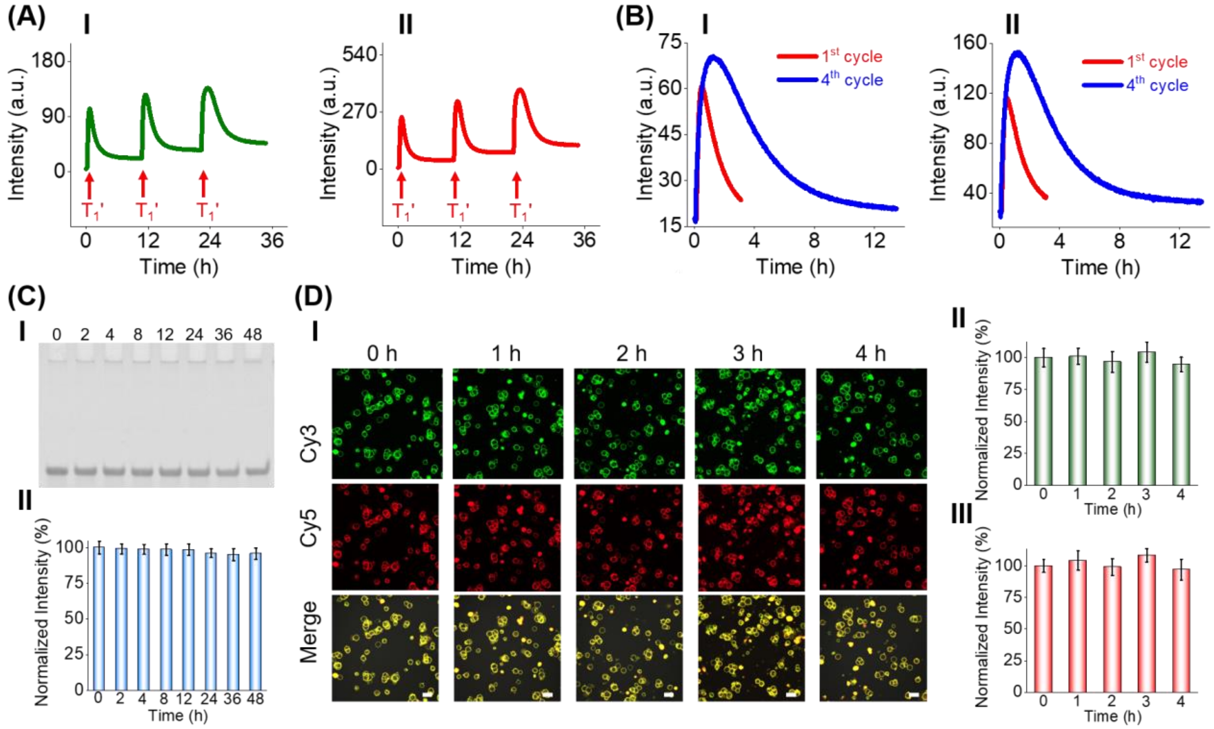

Figure S17. (A) Cyclic T_1'-triggered, transient, temporal concentrations of constituents (CC_1+CD_1), Panel I, and constituents (DC_1+DD_1), Panel II, upon repeated T_1'-triggered activation of evolution/depletion cycles of CDN "M", in culture medium. (B) Transient curves of constituents (CC_1+CD_1), Panel I, and constituents (DC_1+DD_1), Panel II, corresponding to the first cycle adding T_1', and the 4th cycle adding T_1'. (C) Panel I-Gel electrophoresis analysis (native) of stability of the constituents in CDN "M" in culture medium at different time interval, and Panel II-the corresponding quantification analysis of gel electrophoresis. (D) Panel I-Temporal confocal fluorescence microscopy images corresponding to fluorophore Cy3 (green) associated with the constituents (CC_1+CD_1), fluorophore Cy5 (red) associated with the constituents (DC_1+DD_1), and bright-field overlay (yellow), in culture medium. Scale bar, 20 μm. The derived integrated confocal fluorescence intensities of Cy3 (Panel II) and Cy5 (Panel III) derived from Figure S17D, Panel I.

Figure S17 addresses the reusability and stability of CDN "M", displayed in Figure 2, in the presence of the cell culturing medium. Figure S17A depicts the transient fluorescence changes of the constituents CC_1 + CD_1 (Panel I) and DC_1 + DD_1 (Panel II) upon the T_1'-fueled three-cycle operation of the network. Figure S17B, Panels I and II compare the magnified dynamic dissipative, transient fluorescence curves corresponding to CC_1 + CD_1 and DC_1 + DD_1, respectively (1st and 4th transient cycles). The results demonstrate the recyclability of the network. Nevertheless, the transient temporal recovery of the network to its parent state becomes longer as the increase of cycling times. This might be attributed to the dilution of the sample due to the addition of the fuel T_1'. The dilution of the system is expected to dilute nickase concentration and thereby prolong the dissipative process. (Other factors affecting the recyclability could involve minute deactivation of

nickase or the generation of the waste products T_{1-1}' and T_{1-2}' that accumulate and hybridize with T_1, thereby slowing the recovery of the parent state.)

Moreover, Figure S17C, Panel I, shows the gel electrophoresis of constituent CC_1 of CDN "M" associated with the HEK-293T cells, and Panel II depicts the integrated stained band intensities. Evidently, no degradation products was observed, and the intensities of the stained bands are unchanged, demonstrating the stability of the network. Furthermore, Figure S17D, Panel I, shows the confocal microscopy images of parent DNA module associated with the HEK-293T cells prior to the evolved CDN "M". Figure S17D, Panels II and III, depicts the integrated fluorescence intensities associated with constituents C and D, respectively, along a time duration of 4 h. Evidently, the fluorescence intensities of the constituents are unchanged during this time-interval, demonstrating the stability of the constituents on the cell membrane. It should be noted that all *in vitro* cell experiment used FBS-free culture media at 37 °C, in order to avoid FSB-induced degradation of the synthetic DNA frameworks and to minimize cell proliferation that might perturb the composition of the nucleic acid networks associated with the cell membranes.

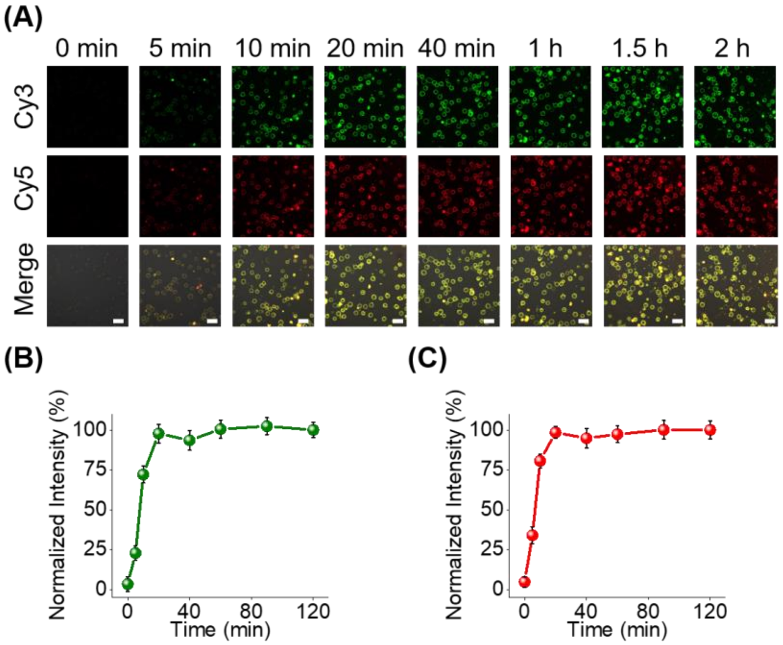

Figure S18. (A) Temporal confocal fluorescence microscopy images corresponding to fluorophore Cy3 (green) associated with the constituents (CC_1+CD_1), fluorophore Cy5 (red) associated with the constituents (DC_1+DD_1), and bright-field overlay (yellow), in culture medium in the presence of the fuel strand T_1' yet in the absence of nicking enzyme. Scale bar, 20 µm. The derived integrated confocal fluorescence intensities of Cy3 (B) and Cy5 (C) derived from Fig. S18A.

Moreover, the stability of the dynamically reconfigured CDN "M" on the HEK-293T cell membrane, in the absence of nickase was probed, Figure S18, by following the temporal fluorescence intensities of Cy3 and Cy5 in constituent (CC_1+CD_1) and (DC_1+DD_1) using the two fluorescence channels. Figure S18A depicts the temporal fluorescence intensities associated with the cells, and Figure S18B and S18C depicts the integrated fluorescence intensities of the two fluorophores upon the temporal reconfiguration into CDN "M". Evidently, upon the triggered formation of CDN "M" in the absence of nickase, the fluorescence intensities of Cy3 and Cy5 reached saturated values at about 20 min, and remained unchanged along the time interval of 2 h, implying that the constituents of CDN "M" modified on the cell membrane are stable in the absence of nickase, without dissociation for at least 2 h.

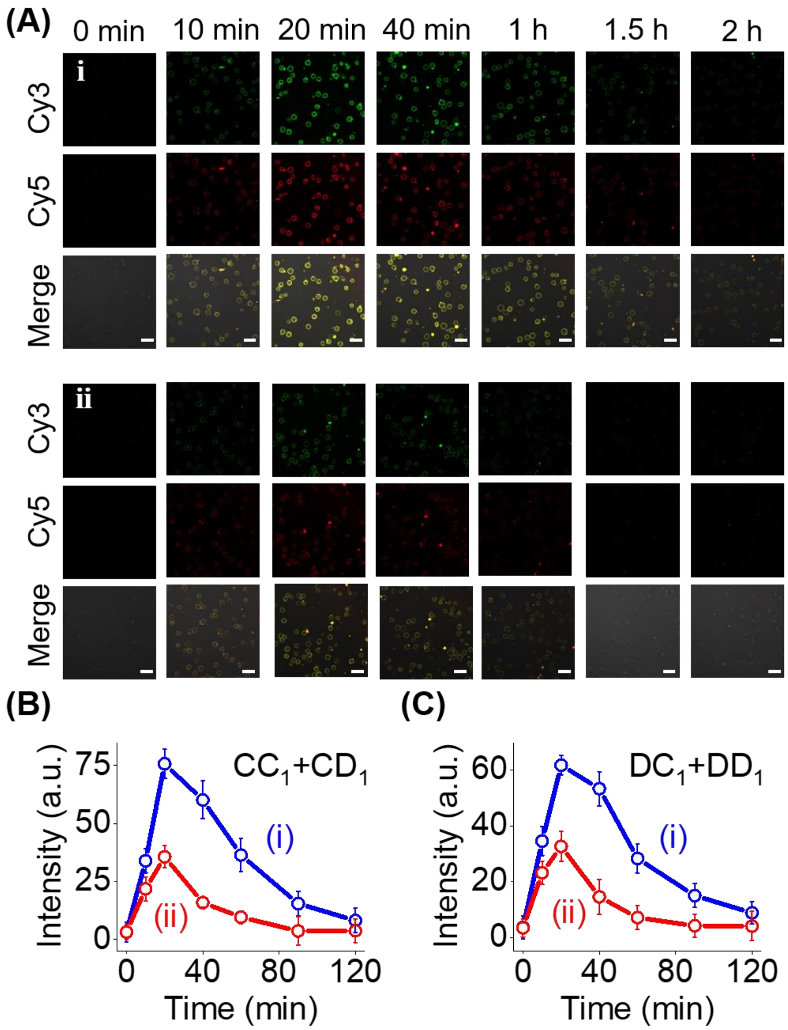

Figure S19. The effect of concentration of nicking enzyme on the dissipative evolution of CDN "M" on the HEK-293T cell membrane. (A) Fluorescence microscopy images of dissipative networks on cell membrane before (t = 0) and after different time intervals following the addition of different concentrations of nicking enzyme at a fixed concentration of fuel T_1', scale bar is 20 μm. The mean fluorescence intensities of Cy3 (B) and Cy5 (C) obtained from fluorescence microscopy images derived from Figure S19A.

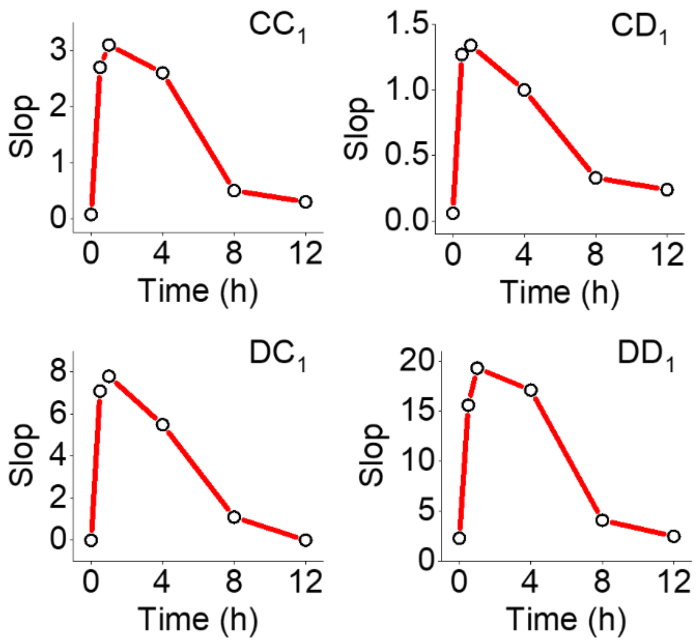

Figure S20. Transient dissipative catalytic rates of DNAzyme during T_1'-fueled dissipative evolution of CDN "M" in culture medium. Time-dependent catalytic rates of DNAzyme units associated with the constituents during T_1'-fueled dissipative evolution of CDN "M" Time-dependent catalytic rates of DNAzyme units associated with the constituents during T_1'-fueled dissipative evolution of CDN "M" at a fixed concentration of fuel T_1', 5 μM and nicking enzyme, 0.046 μM in culture medium.

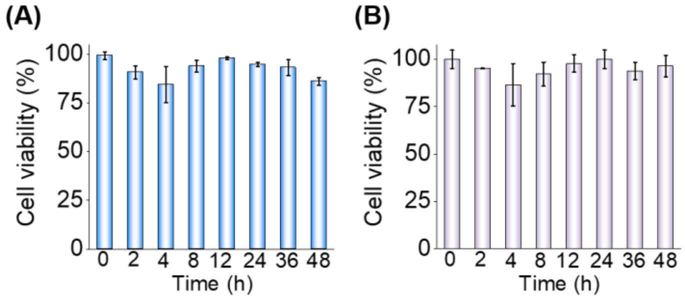

Figure S21. Relative cell viability of HEK-293T (A) and MCF-7 cells (B) subjected to the DNA circuits at different time-intervals.

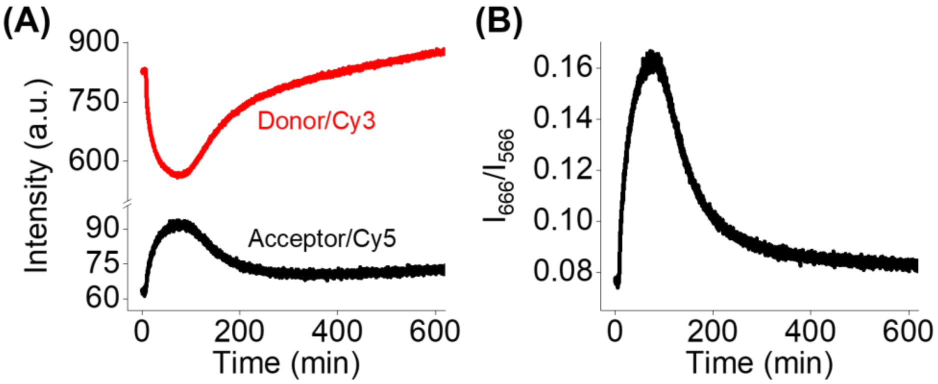

Figure S22. Transient dissipative FRET intensity corresponding to T_1'-fueled dissipative evolution of CDN "N" in culture medium. (A) Time-dependent dissipative fluorescence changes of the donor Cy3 (red) and acceptor Cy5 (black). (B) Time-dependent dissipative FRET intensity ratio of the acceptor and donor, I_{666}/I_{566}, which is derived from Figure S22A. The network module consists of E_1/T_1, E/Q_1, F_1/T_1, and F/Q_1 (1 μM each), and was operated in the presence of the fuel strand T_1', 5 μM, and the nicking enzyme, 0.092 μM.

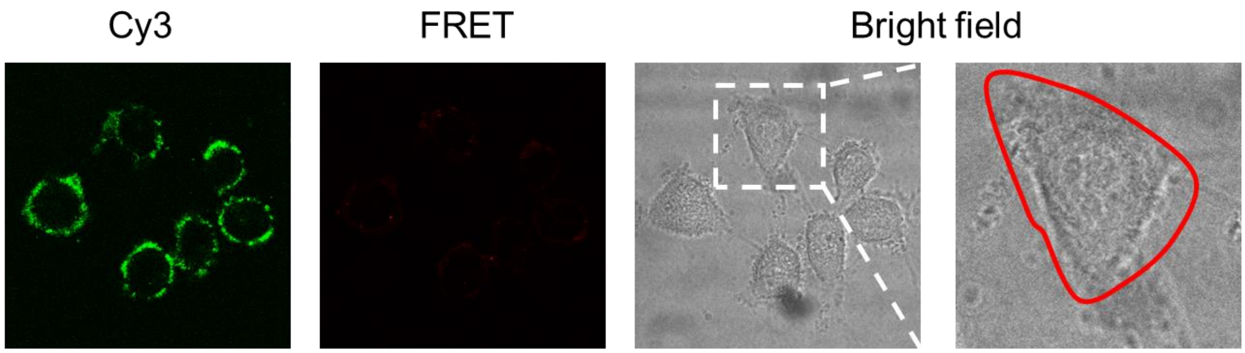

Figure S23. Confocal fluorescence microscopy images corresponding to fluorophore Cy3 (green), FRET signal (red), and bright field high-resolution image associated with the respective CDN constituents on MCF-7 cells, in culture medium.

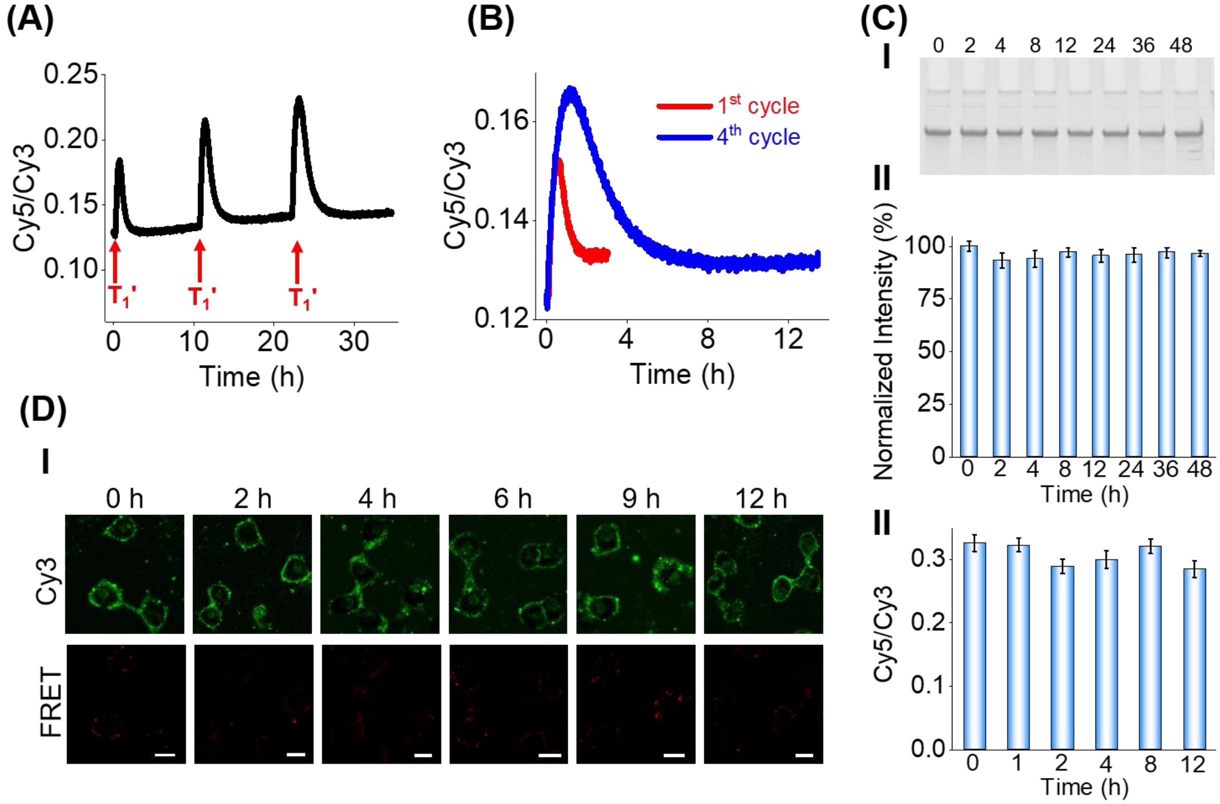

Figure S24. Stability and recyclability of CDN "N" on the MCF-7 cells. (A) Cyclic T_1'-triggered, transient, temporal FRET signal of Cy3/Cy5 fluorophores associated with the respective CDN constituents shown in Figure 3A, upon repeated T_1'-triggered activation of evolution/depletion cycles of CDN "N", in culture medium. (B) Transient dissipative curves corresponding to the resetting of the dissipative system shown in Figure 3(A): Transient curves corresponding to the first cycle adding T_1' and to the 4th cycle adding T_1'. (C) Panel I-Gel electrophoresis analysis of stability of the constituents in CDN "N" in culture medium at different time interval, and Panel II-the corresponding quantification analysis of gel electrophoresis. (D) Panel I-Temporal confocal fluorescence microscopy images corresponding to fluorophore Cy3 (green) and fluorophore Cy5 (red) in culture medium. Scale bar, 20 μm. The derived integrated confocal fluorescence intensities of Cy5/Cy3 (Panel II) derived from Panel I.

The stability of CDN "N" on the MCF -7 cells and the capacity to recycle the transient operation of the network on the cells is a major issue to consider. Figure S24A shows the transient FRET signals of Cy3/Cy5 generated by three repeated T_1'- driven CDN "N" cycles. Figure S24B compares the transient FRET signal after the operation of the 4th cycle in comparison to the 1st cycle. As the number of cycles increased, the peak FRET signal is higher and the transient recovery of the parent reaction module is prolonged. These phenomena can be attributed to a dilution of the system upon recycling that decrease the effective concentration of nickase, and to the accumulation of the "waste" products $T_{1\text{-}1}'$ and $T_{1\text{-}2}'$ that hybridize to T_1, thereby slowing the transient process.

Nevertheless, the results demonstrate the stability and recyclable capacities of the system. Figure S24C, Panel I depicts the native gel electrophoretic analysis, at time-intervals, of constituent EE_1 present in the cell culture medium along 48 h. Evidently, no degradation products is observed. Figure S24C, Panel II shows the quantitative analysis of the respective electrophoretic bands. The results demonstrate the stability of the constituents within this time interval. Furthermore, the stability of the parent reaction module associated with the MCF-7 cells, prior to the T_1'-guided reconfiguration into CDN "N" was probed, Figure S24D. As the constituents F/Q_1 and F_1/T_1, are labeled with Cy3 and Cy5 fluorophore, the entire composition of four constituents was integrated with the MCF-7 cells, and the fluorescence intensity of the system was probed along 12 h. The temporal confocal fluorescence microscopy images of the systems through the Cy3 and Cy5 channels are displayed in Figure S24D, Panel I. The temporal integrated fluorescence intensities of Cy5/Cy3 along 12 h are presented in Panel II. Evidently, minute changes in the fluorescence intensities over time are observed, indicating that the constituent assembly on the cell membrane is stable for at least 12 h.

Control experiments supporting the transient CDN "N"-guided Met-dimer signaling of the p-AKT/FAK pathway

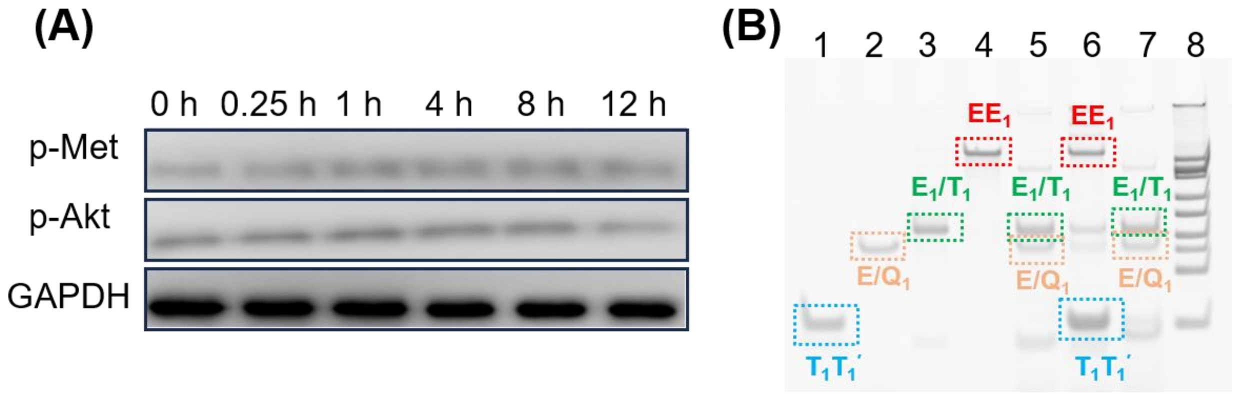

Figure S25. (A) Western Blots corresponding to the time-dependent analysis of phosphorylated Met (p-Met) and phosphorylated Akt (p-Akt) in MCF-7 cells. GAPDH is employed as internal control standard that does not participate in the dynamic signaling network. (B) Gel electrophoresis (native) demonstrating T_1'-fueled dissipative evolution of dimer EE_1. Lane 1 - T_1T_1'; lane 2 - E/Q; lane 3 – E_1/Q; lane 4 - E_1/T_1; lane 5 - EE_1; lane 5 - the original system before subjecting the system to T_1'; lane 6 corresponding to the dimer EE_1 upon the addition of T_1' to the dissipative system for a time interval of 30 min; lane 7 - corresponding to the separated constituents after a time interval of 12 h following the addition of T_1' to the dissipative system.

Figure S25A depicts the Western Blots corresponding to the temporally analyzed phosphorylated p-Met and p-Akt in MCF-7 cell samples without CDN components. Evidently, under these conditions, no p-Met or p-Akt are observed, indicating that the T_1' fuel triggered reconfiguration of the constituents to CDN "N" accompanied by Met dimer is, indeed essential to signal the phosphorylation pathway.
